# Forging a New Frontier: Antimicrobial Peptides and Nanotechnology Converging to Conquer Gastrointestinal Pathogens

**DOI:** 10.1002/smll.202501431

**Published:** 2025-05-19

**Authors:** Christian Shleider Carnero Canales, Cesar Augusto Roque‐Borda, Jessica Ingrid Marquez Cazorla, Renzo Marianito Marquez Cazorla, Uner Josseph Pinto Apaza, Vanderson de Jesus Silva, Laura Maria Duran Gleriani Primo, Maura Jennifer Martínez‐Morales, Rafael Miguel Sábio, Hélder A. Santos, Fernando Rogério Pavan

**Affiliations:** ^1^ Vicerrectorado de Investigación Universidad Autónoma del Perú Lima 150142 Perú; ^2^ School of Pharmacy Biochemistry and Biotechnology Santa Maria Catholic University (UCSM) Arequipa 04013 Perú; ^3^ Tuberculosis Research Laboratory, School of Pharmaceutical Science São Paulo State University (UNESP) Araraquara 14800‐903 Brazil; ^4^ Laboratoire de Chimie des Polymères Organiques Université de Bordeaux Bordeaux 33000 France; ^5^ Department of Biomaterials and Biomedical Technology The Personalized Medicine Research Institute (PRECISION) University Medical Center Groningen University of Groningen Groningen 9713 AV The Netherlands

**Keywords:** antimicrobial peptide, gastrointestinal infections, multidrug resistance, nanocarriers, nanoparticles

## Abstract

Gastrointestinal infections, which are caused primarily by pathogenic bacteria, remain a significant global health challenge. Their resilience is reinforced by various physical, biological, and biopharmaceutical barriers that complicate conventional therapeutic strategies. This review delves into the intricate landscape of managing these infections, addressing microbiota imbalances, the emergence of multidrug‐resistant strains, and the impact of dysbiosis and antibiotic overuse. Faced with these challenges, traditional therapies often fail, which is hindered by low bioavailability, prolonged regimens, and a growing risk of resistance. In this context, nanotechnology applied to antimicrobial peptides (AMPs) has emerged as a promising solution to enhance their stability and targeted delivery. Through a critical approach, diverse nanocarriers and their efficacy against intestinal pathogens are evaluated both in vitro and in vivo. This review advocates for intensified research on the encapsulation and functionalization of AMPs, envisioning their potential to redefine the control of intestinal infections on a global scale.

## Graphical abstract

This review explores the potential of antimicrobial peptides (AMPs) and nanotechnology for the treatment of gastrointestinal infections. It analyzes bacterial resistance mechanisms, the antimicrobial actions of AMPs, and advances in nanocarriers, such as lipid‐based, polymeric, metallic, and mesoporous silica systems, that enhance peptide protection, targeting, and release. Current preclinical strategies and future perspectives toward clinical translation are discussed.

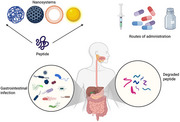

## 1 Introduction

Intestinal infections, triggered by the invasion and uncontrolled proliferation of pathogenic bacteria in the gastrointestinal tract (GIT), pose a significant challenge to human health.^[^ ^1^, ^2^ ^]^ These infections not only affect an individual's immediate well‐being but also impact on the composition and functionality of the gut microbiota.^[^ ^3^ ^]^ The microbiota, a complex community of microorganisms cohabiting in the gut, plays a crucial role in digestive system homeostasis and in regulating immune responses.^[^ ^4^ ^]^

The balance between beneficial and potentially harmful bacteria in the gut microbiota is essential for maintaining intestinal and systemic health.^[^ ^5^ ^]^ Bacterial infections disrupt this balance, triggering inflammatory responses and altering the taxonomic composition of the microbiota. This phenomenon affects gut function and has been linked to systemic consequences, including metabolic, immunological, and neurological changes.^[^ ^4^ ^]^

As our understanding of the complex interactions between intestinal infections and the microbiota advances, a critical need arises to address the infectious aspects of these diseases and their long‐term implications for host health.^[^ ^6^ ^]^ Research in this field seeks innovative therapeutic strategies to combat intestinal bacterial infections and approaches to restore microbial homeostasis and promote holistic recovery of the GIT system. Such advancements could revolutionize the management of intestinal infections, opening new horizons for understanding and treating various diseases associated with microbial dysbiosis.^[^ ^7^ ^]^

Given this background, AMPs have emerged as promising therapeutic agents against intestinal infectious bacteria, including notable pathogens such as *Escherichia coli* and *Salmonella* sp.^[^ ^8^, ^9^, ^10^, ^11^ ^]^ AMPs exhibit antimicrobial effects through multiple mechanisms, interfering with bacterial cell membranes, disrupting essential intracellular processes, and modulating immune responses to enhance host defenses, adding to their therapeutic appeal.^[^ ^12^ ^]^ Owing to their diverse mechanisms of action, these peptides typically exhibit a reduced rate of resistance compared to conventional antibiotics. However, bacterial resistance mechanisms against antimicrobial peptides have been reported, emphasizing the importance of rational peptide design to maintain their therapeutic efficacy and specificity against intestinal pathogens.^[^ ^13^, ^14^ ^]^

In the context of increasing resistance to conventional antibiotics, ongoing research in this area seeks to leverage the distinctive properties of AMPs to provide effective alternatives for the control of intestinal infections.^[^ ^15^ ^]^ On the other hand, AMPs are unstable in biological systems when administered orally, as they pass through the GIT.^[^ ^16^, ^17^ ^]^ Many of the enzymes present in the GIT can hydrolyze or denature AMPs, preventing these molecules from fully reaching the intestinal tract and reducing the ideal treatment concentration to combat pathogenic bacteria in the microbiota.^[^ ^18^ ^]^

Owing to these challenges, nanotechnology has emerged as an innovative tool in the fight against intestinal bacterial infections.^[^ ^19^ ^]^ AMP‐loaded nanoparticles have garnered attention as a potentially effective strategy to address these health challenges. This unique approach leverages the intrinsic antimicrobial properties of AMPs, enabling targeted delivery and protection of AMPs in addition to controlled and sustained release in the GIT.^[^ ^20^ ^]^ The effectiveness of these nanocarriers lies in the ability of nanomaterials to increase the stability, bioavailability, and selectivity of AMPs, thereby increasing their efficacy against intestinal pathogens.^[^ ^9^, ^21^ ^]^ This combination promises to combat intestinal infections more effectively, mitigating potential side effects and minimizing the development of bacterial resistance. In this context, exploring the efficacy of AMP‐loaded nanocarriers represents a significant frontier in the design of innovative therapeutic strategies to address the complexities of intestinal bacterial infections.

## 2 Intestinal Microbiota

The intestinal microbiota is a complex community of microorganisms residing in the GIT that functions as an integrated organ, playing a critical role in host metabolism by facilitating various physiological and biochemical processes.^[^ ^22^ ^]^ It comprises a vast community of nearly 100 trillion microorganisms, including bacteria, archaea, viruses, fungi, helminths, and protozoa.^[^ ^23^, ^24^, ^25^ ^]^ The microbiota represents approximately ten times the total number of cells in the human body, with hundreds of microbial species collectively encoding over 100 times the number of unique genes in the human genome.^[^ ^26^ ^]^

Over 90% of the microbiota consists of bacteria belonging to the *Firmicutes* and *Bacteroidetes phyla*; however, bacteria from the *Proteobacteria*, *Actinobacteria*, *Fusobacteria*, and *Verrucomicrobia phyla* are also present.^[^ ^22^ ^]^ More than 1000 bacterial species have been documented within the microbiota, with estimates suggesting up to 36 000 species in total. Most of these bacteria belong to the genera Bacteroides, Eubacterium, Clostridium, Ruminococcus, and Faecalibacterium, which are strict anaerobes. This trait presents challenges for culturing and characterizing these bacteria; however, the use of selective culture media and multiplex sequencing of conserved 16S ribosomal RNA (rRNA) gene amplicons have advanced our understanding of these species.^[^ ^23^, ^26^ ^]^ In addition to bacteria, the microbiota also includes archaeal species such as *Methanobrevibacter smithii* and *Methanosphaera stadtmanae*; fungi from the phyla Ascomycota (genera Candida and Saccharomyces) and Basidiomycota; and helminths such as cestodes, nematodes, and trematodes.^[^ ^24^, ^27^, ^28^ ^]^

The distribution of the microbiota varies throughout the digestive tract, as each region hosts different microorganisms influenced by unique GIT physicochemical characteristics, such as motility, pH, temperature, redox potential, nutrient supply, ion concentrations, and host secretions.^[^ ^29^, ^30^ ^]^ The microbial density is lowest in the stomach (≈10¹ microbial cells per gram of content), increases in the jejunum and ileum (10⁴–10⁸ cells), and is highest in the colon (10¹^2^–10¹⁴ cells), where approximately 1,100 species can be found.^[^ ^31^, ^32^ ^]^

The microbiota begins developing early in life, with bacterial colonization occurring during birth and breastfeeding. This initial colonization is vital for the immune system and metabolic development.^[^ ^33^, ^34^ ^]^ The infant microbiota has a highly variable and unstable composition but increases in stability and diversity into adulthood.^[^ ^35^ ^]^ Studies have shown that certain genera, such as Bacteroides, are more abundant in women than in men, with body mass index (BMI) also affecting their abundance, e.g., Bacteroides abundance is lower in men with higher BMIs, illustrating the variability in the microbiota between individuals.^[^ ^36^ ^]^ Despite the diversity of the microbiota, a group called the core microbiome is shared by at least half of all adults, comprising fifty taxa dominated by three genera, Bacteroides, Prevotella, and Ruminococcus, independent of age, sex, nationality, or BMI.^[^ ^37^, ^38^ ^]^

The intestinal microbiota plays essential roles in metabolic, physiological, nutritional, and immunological processes, such as supporting the development of intestinal microvilli; facilitating intestinal maturation; facilitating nutrient extraction; regulating secretion and motility; stimulating angiogenesis; providing pathogen resistance; regulating fat storage; and modulating innate and adaptive immunity.^[^ ^24^, ^39^ ^]^ Furthermore, the microbiota limits the resources available to pathogenic microorganisms, acting as a barrier to limit access for nonconsortium members.^[^ ^40^ ^]^

This mutualistic relationship benefits both the microbiota and the host. The microbiota thrives in a warm, nutrient‐rich gut environment, whereas humans benefit from enhanced digestion, nutrient absorption, increased vitamin availability, and pathogen resistance.^[^ ^41^, ^42^ ^]^ However, diet plays a significant role in shaping microbiota composition and colonization, as it supplies essential nutrients for both the host and microbiota. Additionally, the microbiota provides enzymes to break down food components, such as polysaccharides, starch, lignin, collagen, and elastin, that the human body cannot digest alone.^[^ ^33^, ^43^ ^]^

The microbiota also produces critical nutrients that humans cannot synthesize, such as short‐chain fatty acids, vitamins (e.g., vitamin K, vitamin B12, and folic acid), and amino acids.^[^ ^41^ ^]^ For example, a high‐fat, high‐protein diet increases the presence of bile‐tolerant bacteria such as Alistipes, Bilophila, and Bacteroides, whereas a diet rich in starch promotes the growth of *Ruminococcus bromii* and *Eubacterium rectale*, which are known for their saccharolytic properties.^[^ ^44^, ^45^ ^]^ While the microbiota offers numerous benefits, its containment within the intestinal lumen is essential to prevent its invasion into other host tissues, which could cause disease. Several mechanisms, such as intestinal motility, mucin secretion by goblet cells, chloride secretion, AMPs, defensins, and the epithelial barrier, prevent bacterial and antigen passage.^[^ ^46^, ^47^ ^]^

However, microbiota composition can change due to factors such as diet, age, stress, infections, host metabolism, hormonal shifts, environmental influences, or antibiotic use, disrupting the balance of native organisms and promoting pathogenic growth—a phenomenon known as dysbiosis. Dysbiosis is associated with a range of diseases, ranging from neurological and respiratory disorders to metabolic, hepatic, cardiovascular, and gastrointestinal issues, including inflammatory bowel disease (IBD), Crohn's disease, cancer, and ulcerative colitis.^[^ ^48^ ^]^ Dysbiosis has also been linked to immune‐mediated extraintestinal diseases, such as rheumatoid arthritis, multiple sclerosis, diabetes, atopic dermatitis, asthma, obesity, and metabolic syndrome.^[^ ^22^ ^]^ In patients with ulcerative colitis, dysbiosis is characterized by reduced levels of butyrate‐producing bacteria, such as *Faecalibacterium prausnitzii* and *Roseburia hominis*.^[^ ^49^, ^50^ ^]^

Additionally, during dysbiosis and obesity development, the microbiota composition shifts, leading to an increased Firmicutes/Bacteroidetes ratio.^[^ ^51^ ^]^ Dysbiosis of the gut microbiota is closely associated with colorectal cancer (CRC), which is characterized by elevated populations of Streptococcus bovis, enterotoxigenic Bacteroides fragilis, Fusobacterium nucleatum, Enterococcus faecalis, *E. coli*, and Peptostreptococcus anaerobius, which are linked to CRC progression.^[^ ^40^ ^]^

Therefore, the scientific evidence presented above has demonstrated a correlation between dysbiosis and various metabolic and inflammatory diseases, due to its direct involvement in the systemic pathophysiology of the organism. This microbial imbalance arises from the proximity between the gut microbiota and the colonic mucosa. When this relationship is disrupted during dysbiosis, commensal bacteria are transformed into pathogenic bacteria, breaking intestinal homeostasis.^[^ ^52^ ^]^ The study of interactions between the microbiota and the host is essential for the development of therapeutic strategies aimed at restoring eubiosis.^[^ ^53^ ^]^

### 2.1 Bacterial‐Mediated Immune and Inflammatory Responses

Dysbiosis reflects alterations in the microbial community, affecting bacterial proportions and activating the immune response, especially when pathogenic bacteria begin to proliferate.^[^ ^46^ ^]^ The immune system, which is composed of innate and adaptive immune cells, is responsible for balancing pathogen elimination while avoiding harmful responses to beneficial microbiota organisms.^[^ ^54^ ^]^ The first line of defense is the intestinal epithelial barrier, where mucin in the submucosa—a glycoprotein polymer secreted by goblet cells—restricts bacterial adhesion. Similarly, Paneth cells release granules, and intestinal epithelial cells secrete AMPs such as defensins, cathelicidins, and C‐type lectins.^[^ ^43^ ^]^ When this barrier is breached by pathogens, additional defense mechanisms are triggered, including the activation of intraepithelial lymphocytes with TLR receptors, initiating a cascade of signaling pathways. These pathways involve interferon response factors, mitogen‐activated protein kinases, and NFκB, leading to proinflammatory cytokine secretion^[^ ^47^ ^]^

Additionally, immune cells such as dendritic cells located below Peyer's patches phagocytize bacteria that cross the epithelium, migrating to mesenteric lymph nodes and inducing B cells to differentiate into plasma cells that secrete IgA, which protects the intestinal epithelium from enteric toxins and pathogens.^[^ ^42^, ^43^ ^]^ When bacteria cross through M cells, activated dendritic cells promote the differentiation of naive CD4 T cells into Treg and TR1 cells, which secrete anti‐inflammatory cytokines such as transforming growth factor (TGFβ) and IL‐10. Additionally, Th1, Th2, and Th17 cells are activated, characterized by the secretion of proinflammatory cytokines such as IFNγ, IL‐4, IL‐5, IL‐13, IL‐23, and IL‐17F.^[^ ^43^ ^]^

Another key defense mechanism is inflammasome activation, an essential innate immune pathway that prevents pathogen invasion by inducing pyroptosis and the secretion of the proinflammatory cytokines IL‐1β and IL‐18.^[^ ^55^ ^]^ Upon bacterial or helminth stimulation, several cytokines—including IL‐25, IL‐33, IL‐1 family cytokines, IL‐6, IL‐8, and anti‐inflammatory cytokines—are secreted.^[^ ^56^ ^]^ When pathogenic bacteria are detected, granulocyte‒macrophage colony‐stimulating factor (GM‐CSF) is secreted, and symbiotic bacteria promote TGFβ expression by epithelial and dendritic cells.^[^ ^57^ ^]^

## 3 Gastrointestinal Infections

Foodborne diseases lead to high morbidity and mortality in both developed and developing countries, with over 200 diseases transmissible through contaminated food by bacteria, viruses, protozoa, and other agents.^[^ ^58^ ^]^ Gastrointestinal infections pose a significant challenge to global health, with high morbidity and mortality rates, particularly in developing countries. According to the World Health Organization, ≈1.7 billion cases of diarrhea occur each year, leading to ≈525 000 child deaths.^[^ ^59^ ^]^

**Figure 1:**
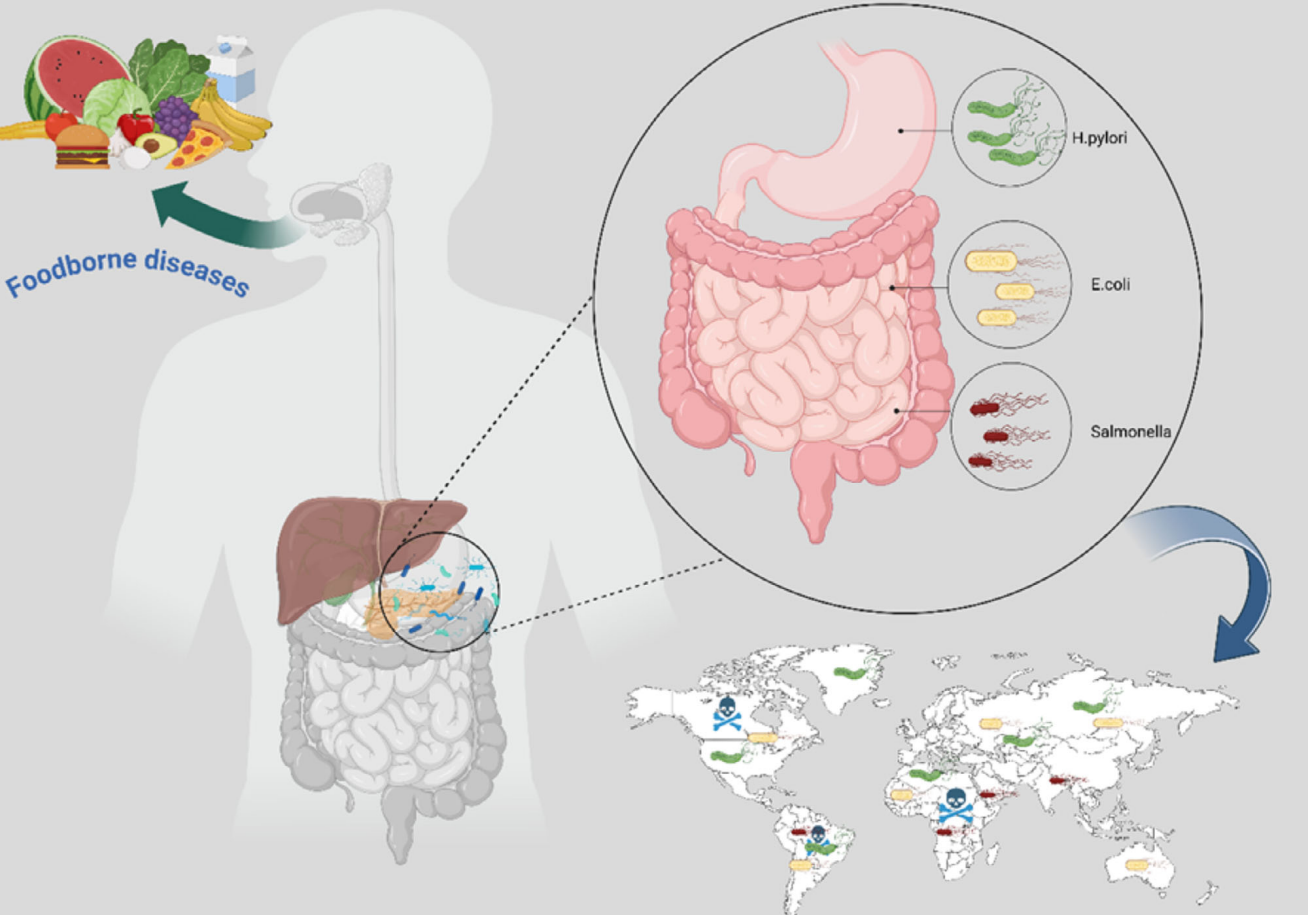
Schematic representation of the main foodborne pathogens (*E. coli*, *H. pylori*, and Salmonella) affecting the human digestive system. The figure illustrates the ingestion of contaminated food, the specific colonization sites of each pathogen within the GIT (stomach and intestines), and a global map highlighting the regions with the highest disease burden and mortality associated with these infections.

Among the most relevant bacterial pathogens in gastrointestinal infections are *H. pylori*, Salmonella spp., and *E. coli*, whose impacts on public health have been exacerbated by the sustained increase in antibiotic resistance (**Figure** **1**).^[^ ^60^ ^]^ *H. pylori*, classified as a class I carcinogen, affects approximately half of the global population, and in some developing countries, its prevalence exceeds 90%. It is an etiological factor for gastritis, peptic ulcers, and gastric carcinoma, and its high prevalence has driven the use of various therapeutic regimens based on proton pump inhibitors combined with antibiotics.^[^ ^61^ ^]^ The emergence of drug‐resistant strains—such as those resistant to clarithromycin, metronidazole, or levofloxacin—has reduced the efficacy of first‐line treatments.^[^ ^62^ ^]^ Between 2011 and 2022, the global prevalence of this bacterium remained alarming, with a rate of 43.1% and levels surpassing 70% in African regions.^[^ ^63^ ^]^

*Salmonella* spp. causes ≈131 million cases of salmonellosis annually, with a high proportion of hospitalizations and mortality in vulnerable populations.^[^ ^64^ ^]^ In addition, the emergence of strains producing extended‐spectrum β‐lactamases (ESBLs) is increasingly complicating outbreak containment.^[^ ^65^, ^66^ ^]^ Historically, the treatment of these infections has involved the use of chloramphenicol, then ampicillin and trimethoprim/sulfamethoxazole, followed by cephalosporins and fluoroquinolones.^[^ ^67^ ^]^ Currently, fluoroquinolones are the drug of choice, although mutations in the gyrA and/or parC genes can compromise their efficacy,^[^ ^68^ ^]^ and plasmids harboring resistance genes also pose a problem.^[^ ^69^ ^]^

With respect to *E. coli*, there are pathogenic variants that cause both intestinal and extraintestinal infections (e.g., urinary tract infections and hemolytic uremic syndrome), displaying a remarkable ability to acquire and disseminate resistance genes, including those encoding ESBLs, carbapenemases, and quinolones.^[^ ^70^ ^]^ For quinolone resistance, mutations in gyrA and parC (the quinolone resistance‐determining region, QRDR) play crucial roles in conjunction with plasmid‐mediated mechanisms.^[^ ^71^ ^]^

Moreover, the epidemiology of *Shigella* depends largely on the geographic region: S. *flexneri* predominates in low‐ and middle‐income countries, *S. sonnei* in high‐income countries, *S. dysenteriae* (serotype 1) is often associated with pandemics in areas affected by natural disasters, and *S. boydii* is observed less frequently.^[^ ^72^ ^]^ First‐line treatment usually involves ciprofloxacin, followed by ceftriaxone and azithromycin.^[^ ^73^ ^]^ However, multiple genes involved in resistance (e.g., blaCTX, mph, ermB, qnr, and mcr) have been identified, conferring resistance to antibiotics such as ciprofloxacin, cephalosporins, azithromycin, and colistin, which are primarily spread via plasmids.^[^ ^74^ ^]^ Each year, Shigella causes ≈200 000 deaths, particularly among children under five years of age in low‐income countries.^[^ ^75^ ^]^

In recent years, gastrointestinal tuberculosis (GITB) and Crohn's disease (CD) have shown epidemiological changes that underscore their diagnostic complexity, as both share symptoms (abdominal pain, fever, diarrhea) and exhibit granulomas in histological studies, often leading to misdiagnoses. A study in South Korea indicated that up to 11% of GITB patients were incorrectly diagnosed with CD, and 18% of CD patients were misclassified as having GITB, delaying treatment and increasing the risk of complications.^[^ ^76^ ^]^

The presence of multidrug‐resistant bacteria—such as ESBL‐producing *E. coli*—also complicates clinical practice. Excessive antibiotic use promotes dysbiosis and favors the spread of resistant strains; moreover, a reduction in bile acids (such as ursodeoxycholic acid (UDCA)) increases susceptibility to infections and inflammatory disorders.^[^ ^77^ ^]^ Infections such as cryptosporidiosis remain concerning: in 2016, more than 48,000 deaths and 4.2 million disability‐adjusted life years (DALYs) were reported in children under five years of age.^[^ ^78^ ^]^ In some countries, it is estimated that up to 92.4% of children have had a *Cryptosporidium* infection by the age of two. Moreover, the global prevalence of *H. pylori* remained at 43.1% between 2011 and 2022, affecting over 70% of the population in Africa and thus increasing the risk of ulcers and gastric cancer.^[^ ^79^ ^]^

Among patients with ulcerative colitis treated with vedolizumab or anti‐TNF therapies, *Clostridium difficile* infection (CDI) was documented in 43 cases, 11 of which were severe, with up to 33% experiencing recurrence. The associated costs of CDI in the United States exceed USD 1.2 billion per year, highlighting the urgency of seeking new diagnostic and therapeutic strategies.^[^ ^80^ ^]^

Surveillance from 2023 revealed a significant increase in several foodborne gastrointestinal infections compared with the 2016–2018 annual average, such as campylobacteriosis (IRR = 1.19), Shiga toxin‐producing *E. coli* (STEC) infections (IRR = 1.33), yersiniosis (IRR = 3.43), vibriosis (IRR = 1.69), and cyclosporiasis (IRR = 4.75). In contrast, the incidence of listeriosis, salmonellosis, and shigellosis remained stable. This apparent increase may be due to the growing use of culture‐independent diagnostic tests (CIDTs), which reached 78% in 2023 (46% were diagnosed solely by the CIDT). The reduction and lower success rates in confirmatory cultures hinder the use of isolates for species characterization and resistance mechanism analysis, complicating the interpretation of epidemiological trends. Furthermore, the expansion of FoodNet to the entire state of Colorado increased the representation of the Hispanic and American Indian populations by 8% and the rural population by 10%, covering 53.6 million people, equivalent to 16% of the US population.^[^ ^81^ ^]^

Polymyxin is considered a last‐line agent for treating infections caused by multidrug‐resistant gram‐negative bacteria; however, emerging resistance to this antibiotic jeopardizes the effectiveness of conventional therapies.^[^ ^82^ ^]^ This review examines the use of AMPs as promising alternatives, as their mechanism of action on bacterial membranes reduces the likelihood of resistance development. Nonetheless, their clinical application faces challenges such as low stability, susceptibility to proteolytic degradation, and, in some cases, toxicity. To overcome these barriers, the use of nanocarriers has been proposed, enabling the encapsulation and protection of AMPs, thereby increasing their therapeutic efficacy and in vivo stability while minimizing potential adverse effects.

## 4 Antimicrobial Peptides in Fight Against Gastrointestinal Infections

**Figure 2:**
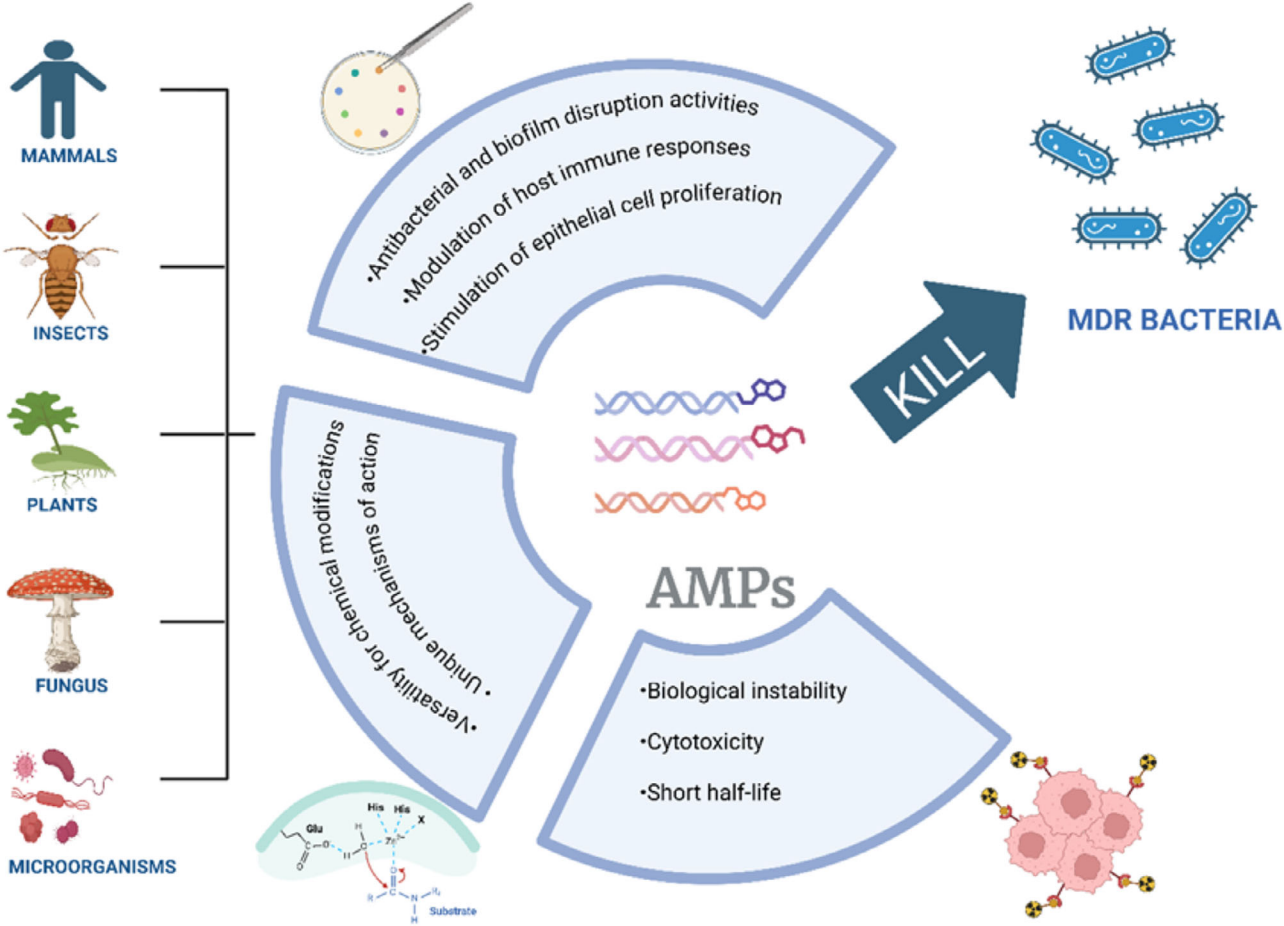
Overview of the origin, biological functions, and current limitations of AMPs in the treatment of MDR bacteria. AMPs are derived from various natural sources, including mammals, insects, plants, fungi, and microorganisms. Their mechanisms of action include antibacterial and antibiofilm activities, modulation of the host immune response, and stimulation of epithelial cell proliferation. However, clinical application is limited by issues such as biological instability, cytotoxicity, and short half‐life.

AMPs are a class of small peptides that are typically composed of 10–60 amino acid residues, are present in the defense systems of various organisms, and are active because of their amphipathic properties.^[^ ^83^ ^]^ They can be found in mammals, insects, plants, fungi, and bacterial species and are secreted in response to disturbances in cellular homeostasis to participate in antimicrobial defense and humoral immunity (**Figure** **2**).^[^ ^84^ ^]^ AMPs are promising compounds because of their multiple applications, including antibacterial and biofilm disruption activities, modulation of host immune responses, stimulation of epithelial cell proliferation, and wound healing.^[^ ^85^, ^86^, ^87^ ^]^ Nevertheless, emerging bacterial adaptations have been reported, as further discussed in Section 5.^[^ ^88^ ^]^

The mechanisms of action of AMPs can be summarized as follows: membrane permeation (using barrel‐stave, toroidal pore, aggregate channel, carpet or sinking raft models and by modifying bacterial cell morphology), biofilm permeation and inhibition (altering biofilm structure through membrane permeation), resensitization of multidrug‐resistant bacteria (enhancing antibiotic efficacy by rapid depolarization of the membrane potential), intracellular bacteriostatic effects (e.g., modifying proton kinetics in ATP synthesis), and immune activity (regulating cytokines, stimulating chemokine release, and neutralizing cellular endotoxins).^[^ ^88^ ^]^ AMPs offer benefits such as slow selection of resistant strains, versatility for chemical modifications, and unique mechanisms of action.^[^ ^89^ ^]^

Among the most studied peptides are defensins and cathelicidins, which are host‐defense cationic peptides that exert bactericidal effects through membrane disruption or inhibition of bacterial transcription and translation. Owing to these varied activities, they induce minimal resistance. The expression of these proteins can be stimulated by inflammatory mediators, such as interleukin (IL)‐1β, as well as by nutrients such as fatty acids. There are two types of defensins, α‐defensins, and β‐defensins, with the latter expressed in epithelial cells of the GIT and activated by proteolytic cleavage. Human cathelicidin (LL‐37) is present in various epithelial cells, such as intestinal epithelial cells, but is more common in neutrophils.^[^ ^90^ ^]^

Bacteriocins, which promote homeostasis, cell proliferation, host immune regulation, and biodiversity control in the GIT, are small, positively charged peptides synthesized by ribosomes. Their mechanism of action is bacterial membrane destruction, especially in gram‐positive bacteria.^[^ ^91^ ^]^ Bacteriocins may cross the gut‒blood barrier, facilitated by their interaction with epithelial cells in the GIT and their size, charge, and hydrophobicity.^[^ ^92^ ^]^

AMPs can be used as therapeutic agents in various ways: as monotherapies for infections, in combination with conventional antibiotics to promote synergistic or additive effects, as immunomodulatory agents to enhance innate immunity, and as endotoxin‐neutralizing agents to prevent fatal complications associated with virulent factors that cause septic shock.^[^ ^93^ ^]^ In a study by Xiong et al.,^[^ ^94^ ^]^ a monotherapy based on transitional antimicrobial polypeptides was developed. These polypeptides, which contain randomly distributed negatively charged glutamic acid residues and positively charged poly(γ‐6‐N‐(methylhexyl ammonium)hexyl‐1‐glutamate), are described by the formula (HCT‐AMP) (PGA)m – r – (PHLG‐MHH)n, where PGA represents negatively charged poly(glutamic acid) and (PHLG‐MHH)n represents positively charged poly(γ‐6‐N‐(methylhexyl ammonium)hexyl‐1‐glutamate). These polypeptides adopt a spiral‐helix conformation with pH‐sensitive bactericidal action and specificity for *H. pylori* under acidic pH conditions, protecting commensal bacteria at physiological pH (pH 7.4).

The in vitro results of Xiong et al.^[^ ^94^ ^]^ revealed that transitional polypeptides exhibited high antimicrobial activity against *H. pylori* under acidic conditions (pH 4) in the stomach, with PL2 demonstrating over 90% efficiency at pH 3 and 4.4 µm. These findings confirmed that monotherapy did not exhibit toxicity toward commensal bacteria at physiological pH because of the pH‐sensitive helical structure of the peptides, which confers specific antimicrobial activity. In vivo results were obtained in mice infected with *H. pylori* SS1 (1 × 10^8^ CFU per animal) via oral gavage. Two weeks after inoculation, the mice were divided into four groups: control (phosphate‐buffered saline (PBS), OAC triple therapy, nonhelical control peptide (PDL2), and transitional peptide (PL2) groups. The results indicated that the bactericidal activity of PL2 was similar to that of the OAC triple treatment and was ≈100 times greater than that of the control. Notably, despite having a similar distribution to that of PL2, PDL2 did not achieve significant antimicrobial activity, highlighting the role of the active helical structure in the membrane in the bactericidal activity against *H. pylori*. Toxicity was evaluated through various assays, including H&E staining, which revealed no significant gastric inflammation. The Terminal deoxinucleotidil transferasa (TUNEL) assay indicated no induced apoptosis in gastric cells and no observed liver or kidney damage. Additionally, PL2 was confirmed to be nontoxic to commensal bacteria, in contrast to OAC treatment, which resulted in 65% and 86% reductions in commensal bacteria in the ileal conduit and feces, respectively. The study concluded that HCT‐AMP peptides with pH sensitivity selectively target *H. pylori* infections in the stomach, with no bactericidal activity at pH 7.4, implying a minimum inhibitory concentration (MIC) exceeding 70 µm under these conditions.

Antivirulence therapies are gaining importance in research, distinguished from antibiotics by their mechanism of action, as they inhibit bacterial virulence factors rather than directly impacting bacterial viability, allowing for greater control and specificity in pathogen virulence.^[^ ^95^ ^]^ An example of this approach is an experimental study by Nodeh et al.,^[^ ^95^ ^]^ where a CPP‐PNA conjugate of 16 nucleotides was used to target cagA virulence gene expression in *H. pylori*. The MTT assay was used to evaluate the viability of cells treated with CPP‐PNA at concentrations of 1, 2, 4, and 8 µmol L^−1^. *H. pylori*‐induced apoptosis was assessed in the RAW 264.7 murine macrophage line under the same CPP‐PNA concentrations. The therapeutic effect was further tested in infected mice treated with CPP‐PNA at 5 mg kg^−1^. The results showed that CPP‐PNA inhibited cagA gene expression in a dose‐dependent manner, with the lowest expression occurring at 8 µM CPP after 6 h, effectively reducing *H. pylori* pathogenicity. Additionally, CPP‐PNA showed minimal cytotoxicity at doses ≤8 µm across both cell lines and did not present detectable toxicity or immunogenicity in experimental animals, suggesting that CPP‐PNA is a promising antisense therapy for *H. pylori*‐induced infections in the stomach.

One of the most potent AMPs introduced for *H. pylori* elimination is epinecidin‐1, which can inhibit both antibiotic‐sensitive and antibiotic‐resistant *H. pylori* strains, with an MIC of 8 to 12 µg mL^−1^, as reported by Neshani et al.^[^ ^96^ ^]^ This finding aligns with the results of Narayana et al.,^[^ ^97^ ^]^ who demonstrated that oral administration of epinecidin‐1 (250 µg) fully eliminated *H. pylori* from the stomach in C3H/HeN mice without adverse toxicity, which was supported by total blood biochemistry and macroscopic necropsy of vital organs.

Fonseca et al.^[^ ^98^ ^]^ reported that pexiganan is one of the few AMPs capable of completely eradicating *H. pylori*. The authors described the use of chitosan nanoparticles as a protective system for pexiganan against fluctuations in the pH of the stomach and mucosal regeneration, which contribute to peptide degradation. They reported an MIC of 4 µg mL^−1^ in this nanocarrier, as this delivery method's bioadhesive properties in the stomach enhance pexiganan's antimicrobial activity by allowing the peptide to remain adhered in the stomach longer.

Wang et al.^[^ ^99^ ^]^ evaluated the antimicrobial and membrane activity of HJH‐1, a cationic peptide derived from bovine erythrocyte hemoglobin α‐subunit P3. The MICs were 12.5 and 6.25 µg mL^−1^ for *E. coli* and clinical Salmonella, respectively. HJH‐1 was stable at high temperatures, and its activity was unaffected by pH changes. HJH‐1 exhibited low hemolytic activity against rabbit red blood cells, even at 400 µg mL^−1^ (>5 × MIC), causing less than 20% hemolysis. In later studies, Wang *et al.* ^[^ ^100^ ^]^ designed and synthesized a series of HJH‐1‐derived peptides, with HJH‐5 and HJH‐6 exhibiting the highest activity against the tested strains. The MIC for HJH‐5 and HJH‐6 was 1.5 µmol for both *E. coli* and *S. aureus*. HJH‐5/HJH‐6 also showed slight cytotoxicity against human blood cells and normal cell lines, indicating that hydrophobic residues and helices contribute to enhanced antimicrobial activity, hemolytic activity, and cell viability.

The synthetic model of bacteriocins differs from that of antibiotics, as bacteriocins are produced through mRNA translation and generally exhibit broad‐spectrum bacterial activity with high effectiveness at nanomolar concentrations. In one study, the bacteriocin BM1829 effectively eliminated *E. coli* and S. aureus, with MIC values of 1 and 16 mg L^−1^, respectively.^[^ ^101^ ^]^ In accordance with Naimi et al.,^[^ ^102^ ^]^ microcin J25 (MccJ25), a bacteriocin produced by *E. coli*, was analyzed for its bactericidal effect against Escherichia, Salmonella, and Shigella pathogens via the TIM‐1 dynamic simulator model of the GIT. Simulated digestion was conducted with a sterile MccJ25 solution at 0.1 mg mL^−1^. Despite the stable structure of MccJ25, duodenal proteases degrade it, limiting its bactericidal activity. Antibacterial activity, measured at t0 and after 30 and 60 min in gastric conditions, decreased from 16,384 AU mL^−1^ to 4,096 AU mL^−1^ upon exposure to duodenal conditions, which is a limiting factor for oral administration; therefore, protective technology is necessary to maintain its antimicrobial activity.

Polyudova et al.^[^ ^103^ ^]^ demonstrated the sensitivity of *E. coli* to nisin (Nisaplin) and staphylococcal peptides (warnericin and hominidin) under osmotic stress with sucrose. At 1 mg mL^−1^ of each peptide, no growth inhibition was observed without sucrose. However, with 0.3 g mL^−1^ sucrose and 1 mg mL^−1^ Nisaplin, a reduction of up to 3 logs in *E. coli* cells occurred. These results suggest that sucrose enhances the bactericidal activity of peptides, particularly nisin.

Combining antibiotics with AMPs can enhance their bactericidal effect. Ridyard et al.^[^ ^104^ ^]^ evaluated the efficacy of six common antibiotics (including ampicillin, tetracycline, and polymyxin B (PMB)) in combination with AMP LL‐37 against *E. coli* and Pseudomonas aeruginosa. LL‐37 was chosen for its antimicrobial and immunomodulatory activity, as it regulates chemokine and cytokine production, balancing pro‐ and anti‐inflammatory responses.^[^ ^105^ ^]^ Its mechanism is based on altering the cell membrane, leveraging its positive charge (+6) to bind to the anionic membranes of pathogens, causing lysis. Compared with the individual treatments, the combination of PMB and LL‐37 was the most effective, reducing the minimum inhibitory concentration (MIC) of PMB from 1 to 0.25 µg mL^−1^ and improving biofilm inhibition and eradication. These findings suggest that AMP combinations could be a promising strategy for treating infections caused by resistant bacteria.

As a zoonotic pathogenic bacterium, Salmonella is a major cause of gastrointestinal infections, leading to excessive antibiotic use and the failure of standard treatments.^[^ ^103^ ^]^ Recently, Héjja et al.^[^ ^106^ ^]^ characterized a novel AMP isolated from yolk hydrolysate, peptide EYHp6, which inhibited various Salmonella enterica serovars, with MICs of 2 m*M* for *S. typhimurium* TISTR 292 and Enteritidis DMST 15679 and MICs of 4 mM for *S. typhimurium* ATCC 14028 and S. enteritidis ATCC 13076. The mechanism of action is thought to involve membrane destabilization through lipid interactions or alterations in the hydrophobicity of the cell surface. With respect to hemolytic activity, EYHp6 showed no hemolytic effect at its maximum inhibitory concentration (4 mM), making it a potential antibacterial candidate; however, in vivo studies of the intestinal microbiota are needed to ensure its safety.

Wang et al.^[^ ^107^ ^]^ evaluated the effects of the JH‐3 peptide in macrophages infected with Salmonella Typhimurium CVCC541. The importance of macrophages lies in their dual role during infection, as they participate in the immune response and act as host cells for the pathogen, which promotes the spread of infection. This study demonstrated that JH‐3 reduces bacterial growth by inhibiting TLR4 protein expression, decreasing the phosphorylation level of the MAPK signaling pathway (p38), and reducing the activation of the NF‐κB signaling pathway. This inhibition suppresses the expression and secretion of the inflammatory cytokines IL‐2, IL‐6, and TNF‐α, as measured through qRT‒PCR and ELISA.

For anti‐Salmonella peptide development, Magmee et al.^[^ ^108^ ^]^ designed several modified AMPs derived from the BmKn‐2 peptide. The results revealed that variants with cationic amphipathic α‐helical structures exhibited improved antibacterial activity against clinical Salmonella isolates. The peptide Kn2‐5R‐NH₂, with +5 charges and a free carboxyl group at the C‐terminus, showed the highest specificity, with an MIC of 4 µM for both sensitive and resistant strains. These results suggest that increasing positive charges in the peptide enhances the anti‐Salmonella potential because of stronger binding to the negatively charged bacterial surface (phospholipids and polysaccharides). Its hemolytic activity was measured in vitro against sheep red blood cells (sRBCs) at the MIC, with Kn2‐5R‐NH₂ showing an insignificant hemolysis rate of 2%. Based on these results, the Kn2‐5R‐NH₂ peptide can be considered effective and safe, with low toxicity to red blood cells.

Zhao et al.^[^ ^109^ ^]^ described the modification of the OM19R peptide structure. OM19R exhibited antimicrobial activity against gram‐negative bacteria, but it was highly unstable in the presence of trypsin, an enzyme found throughout the digestive tract, which poses a significant obstacle to the oral administration and efficacy of peptides. To address this, L‐arginine and L‐lysine residues were replaced with their corresponding D‐arginine and D‐lysine residues, resulting in a new AMP, OM19D, with improved antimicrobial performance. In sensitive strains of *E. coli*, Salmonella, and Shigella, OM19D had MIC values ranging from 2–4 µg mL^−1^, whereas the MICs of OM19R ranged from 1–2 µg mL^−1^. Additionally, the MICs of both peptides were evaluated in the presence of trypsin, which revealed that OM19D maintained an MIC of 16 µg mL^−1^ even at high trypsin concentrations (10 mg mL^−1^), whereas OM19R had already lost its antibacterial activity at 5 mg mL^−1^. These results suggest that this strategy improves peptide stability against proteases while maintaining their antimicrobial activity. The key highlights of AMPs against these bacteria are summarized in **Table** **1**.

**Table 1:** Applications of AMPs in treating intestinal pathogens.

AMP	Sequence	Activity	MIC	Highlights	Refs.
HJH‐3	VNFKLLSHSLLVTLRSHL	*Salmonella typhimurium, E. coli* and *S. aureus*	3.125,12.5 and 25 µg mL^−1^	It can enter the cell membrane	[110]
sTac‐I	NH_2_‐KWCFRVCYRGICYRRCR	*Listeria monocytogenes*	40 µg mL^−1^	It is a *Limulus* AMP, with linear structure and it lacks disulfide bonds and *β*‐sheets	[111]
OaBac5 mini	RFRPPIRRPPIRPPFRPPFRPPVR	*E. coli*	25 µg mL^−1^	It was synthesized as a truncated fragment of OaBac5; it has potent activity against gram‐negative bacteria but weak activity against gram‐positive bacteria and *C. albicans*	[112]
Amp1D	LKLLKKLLKKLLKLL	MDR *P. aeruginosa*	3.12 µM	It has antibiofilm activity against *P. aeruginosa* Cystic Fibrosis Patients’ Isolate	[113]
Aurein 1.2	GLFDIIKKIAESF	*A. baumannii*	16 µg mL^−1^	It has antibiofilm activity	[114]
Dermaseptin‐AC	GMFTNMLKGIGKLAGKAALGAVKTLA	*E. faecalis*	5.122 µg mL^−1^	It was isolated from the skin secretion of *Agalychnis callidryas*	[100]
K4	KKKKPLFGLFFGLF	*S. aureus*	50 µg mL^−1^	It enhances the bacterial killing mechanism of macrophages	[115]
PMAP‐23	RIIDLLWRVRRPQKPKFVTVWVR	*S. aureus*	0.049 µg mL^−1^	It was isolated from pig bone marrow cells	[116]
CR‐172	RRWVQRWIRRWRPKVAAA ‐RRWVQRWIRRWRPKV	*P. aeruginosa*	10 µM	It can completely neutralize LPS	[117]
Pacavin‐5	FLKKLWKAMKKLL	*E. coli*	6.25 µg mL^−1^	It kills the bacteria by disrupting the membrane	[101]
Kassinatuerin‐3	FIQHLIPLIPHAIQGIKDIF	*E. faecium*	128 µM	It was isolated from the skin secretion *Kassina senegalensis*	[118]
Pln149‐PEP20	Fmoc‐KAVKKLFKKWG	*E. faecium*	16 µg mL^−1^	It has a hemolytic activity of >512 µg mL^−1^	[119]
GW18	GWGAKRWGKRGWKWKRHW	*MRSA‐Z*	1.32 µM	It has low hemolytic activity, cytotoxicity, and no acute toxicity	[120]
D‐BMAP18	GRFKRFRKKFKKLFKKLS‐am (amide group)	MDR *P. aeruginosa*	8‐16 µg mL^−1^	It has anti‐inflammatory activity because downregulated the production of TNF‐α, IL1‐β, and TGF‐β in THP‐1 cells	[121]
Esculentin‐1a(1‐21)NH_2_	GIFSKLAGKKIKNLLISGLKG	*P. aeruginosa*	4 µM	It can downregulate the expression of biofilm‐associated genes of *P. aeruginosa*	[121]
PLG0206	RRWVRRVRRVWRRVVRVVRRWVRR‐	MDR *Acinetobacter baumannii*	1 µg mL^−1^	It has activity against both gram‐positive and gram‐negative MDR ESKAPE	[122]
LyeT I‐bPEG	IWLTALKFLKFLGKNL ‐GKNLGKLAKQQCAKL	*A. baumannii*	16 µM	It has synergistic effects with gentamicin to antibiofilm activity	[123]
AP19	RLFRRVKKVAGKIAKRIWK	*S. typhimurium*	1.95 µg mL^−1^	The D‐enantiomers, maintained antibacterial activity in the presence of proteolytic plasma proteins	[124]
N6	GFAWNVCVYRNGVRVCHRRAN	*E. coli*	4 µg mL^−1^	It is an arenicin‐3 derivative isolated from the lugworm	[125]
As‐CATH7	KRVNWRKVGRNTALGASYVLSFLG	*S. aureus, K. pneumoniae, P. aeruginosa, E. faecium, E. coli* and *S. typhimurium*	4,1,4, >64, 2 and 2 µM	It is a crocCATHs and host defense peptide	[125]

MIC: Minimum inhibitory concentration; *E. coli*: *Escherichia coli*; *S. aureus*: *Staphylococcus aureus*; MDR: Multidrug‐resistant; *P. aeruginosa*: *Pseudomonas aeruginosa*; *A. baumannii*: *Acinetobacter baumannii*; *E. faecalis*: *Enterococcus faecalis*; *E. faecium*: *Enterococcus faecium*; MRSA‐Z: Methicillin‐resistant *Staphylococcus aureus*; *S. typhimurium*: *Salmonella typhimurium*; K. pneumoniae: *Klebsiella pneumoniae*; LPS: Lipopolysaccharide; TNF‐α: tumor necrosis factor‐alpha; IL1‐β: interleukin‐1‐beta; TGF‐β: transforming growth factor‐beta; THP‐1: The human monocytic cell line; ESKAPE: Enterococcus spp, Staphylococcus aureus, Klebsiella pneumoniae, Acinetobacter baumannii, Pseudomonas aeruginosa y Enterobacter spp; crocCATHs: crocodylian cathelicidins

AMPs have been extensively researched for in vivo applications, especially for their role in immune modulation.^[^ ^19^ ^]^ Several antimicrobial peptides, which are currently in the preclinical and clinical phases, have demonstrated promising results in these applications (**Table** **2**). Wu et al.^[^ ^126^ ^]^ investigated the role of dietary cecropin in piglets and broiler chickens. The authors demonstrated that this AMP could increase immunoglobulin A and immunoglobulin G levels from 167.8 µg mL^−1^ and 868.4 µg mL^−1^ to 185.4 µg mL^−1^ and 1020.7 µg mL^−1^ in piglets. Similarly, Bai et al.^[^ ^127^ ^]^ reported that dietary supplementation with cecropin increased the spleen index from 0.95 to 1.27 g kg^−1^ and the thymus index from 4.82 to 5.10 g kg^−1^ in broiler chickens. Furthermore, Ren *et al.* ^[^ ^128^ ^]^ reported that dietary supplementation with a basal diet mixed with 250 mg kg^−1^ antibacterial peptide increased the T‐cell index in peripheral blood from 1.27 to 1.58% and reduced spleen cell apoptosis from 23.13 to 14.50% after 53 days of treatment in weaned piglets.

**Table 2:** New peptides in preclinical, clinical, and recently approved phases.

AMP	Structure	Sequence	Origin	Activity	Evaluation Phase	Refs.
GL13K	13 amino acid AMP	GKIIKLKASLKL‐NH₂	Human salivary glands	*S. aureus*, *P. aeruginosa*	Clinical	[131]
Alyteserin	α‐helix	ILGKLLST AAGLSNL.NH₂	Synthetic	*E. coli*	Clinical	[132]
A3‐APO	Lipopeptide	[(CHEX‐ARG‐PRO‐ASP‐LYS‐PRO‐ARG‐PRO‐TYR‐LEU‐PRO‐ARG‐PRO‐ARG‐PRO‐ARG‐PRO‐VAL‐ARG)₂‐DAB]	Synthetic	*E. coli*, *Salmonella*	Preclinical	[133]
Colistin/Polymyxin E	Lipopeptide	C_52_H_98_N_16_O_13_	Synthetic	gram‐negatives	FDA Approved	[134]
Reltecimod	Mimetic of T lymphocyte receptor cd28	C_46_H_72_N_12_O_15_S	Synthetic	Necrotizing soft tissue infections, peritonitis	Phase III	[135]
Omiganan	Indolicidin analog	C_90_H_132_Cl_5_N_27_O	Synthetic	*E. coli*	Phase I/II	[136]
Polymyxin	Cationic polypeptide	C_55_H_96_N_16_O_13_	Synthetic	*E. coli*	FDA Approved	[137]
Pexiganan	Magainin analog	C_122_H_210_N_32_O_22_	Synthetic	*E. coli*, *H. pylori*	Preclinical	[138, 139]
Surotomicin	Lipopeptide	C_77_H_101_N_17_O_26_	Synthetic	*C. difficile*	Phase I	[140, 141]
Nisin	Cyclic polypeptide	C_143_H_230_O_37_N_42_S_7_	*Lactococcus lactis*	*E. coli*, *H. pylori*	FDA Approved	[142, 143]
Gramicidin	Lipopeptide	C_99_H_140_N_20_O_17_	*Bacillus brevis*	*E. coli*, gram‐negative, gram‐positive	Preclinical	[144]
Magainin	Polypeptide	GIKFLHSGKFGKAFVGEIMS	Derived from African clawed frog (*Xenopus laevis*)	*E. coli*, gram‐negative, gram‐positive	Preclinical	[145, 146]
Buforin I	Polypeptide	C_184_H_318_N_70_O_47_	Isolated from the stomach of the Asian toad (Bufo bufo)	*E. coli*	Preclinical	[147]
Mutacin 1140	Lipopeptide	C_103_H_138_N_28_O_23_S	*Streptococcus mutans*	*C. difficile*, gram‐positive	Preclinical	[148]
VV‐14	Cationic polypeptide	VKWKKVKVWKVVK	Synthetic	*Salmonella*	Preclinical	[149]

AMPs can also modulate the gut microbiota by creating a microbial ecology that suppresses pathogenic microorganisms, increases beneficial microorganisms, and improves intestinal morphology.^[^ ^19^ ^]^ This result was reported by Wan et al.,^[^ ^129^ ^]^ who reported that recombinant plectasin increased the abundance of Bifidobacterium from 4.72 log copies g^−1^ to 5.99 log copies g^−1^ and that of Lactobacillus from 6.27 log copies g^−1^ to 6.90 log copies g^−1^ in the ileum of weaned pigs.^[^ ^130^ ^]^ Similarly, Bai et al.^[^ ^127^ ^]^ confirmed this result by demonstrating the role of sublancin (11.52 mg L^−1^) in increasing the height of the duodenal villus from 910.4 to 1104.0 µm. Additionally, the gut microbiota plays an important role in protecting the human body against pathogens and supporting the digestive process. Paneth cells present in the intestinal epithelium produce various AMPs, such as defensins and cathelicidins, which act in local homeostasis, protect the intestinal barrier, and prevent the development of inflammatory bowel disease (IBD).^[^ ^127^ ^]^

Some resistance mechanisms include degradation by proteases and peptidases, high hemolytic activity, release of inhibitory proteins, or changes in the conformation of the outer membrane of pathogenic bacteria.^[^ ^150^ ^]^ Despite recent advancements showing the promising activity of AMPs against bacteria affecting the GIT, many studies report that peptides tend to be exposed to various limiting factors that can alter their mechanism of action.^[^ ^151^ ^]^

## 5 Bacterial Resistance and Limitations of Antimicrobial Peptides

Despite these advantageous characteristics of AMPs, growing evidence highlights that bacteria can employ various adaptive mechanisms, ultimately reducing susceptibility and the development of resistance to AMPs. This section details the known bacterial resistance mechanisms against AMPs and the limitations that currently hinder their clinical application.

### 5.1 Modification of Membrane Charge and Lipid Composition

One major resistance mechanism involves alterations in membrane surface charges that reduce the initial electrostatic attraction between positively charged AMPs and negatively charged bacterial surfaces. Gram‐positive bacteria, notably *S. aureus*, employ the enzyme multiple peptide resistance factor (MprF) to introduce positively charged lysyl‐phosphatidylglycerol (Lys‐PG) into their cell membrane.^[^ ^151^, ^152^ ^]^ Similarly, Gram‐negative bacteria such as *Salmonella enterica* ^[^ ^153^ ^]^ and *P. aeruginosa* ^[^ ^154^ ^]^ can alter their lipopolysaccharide (LPS) structure through enzymatic modification of lipid A components. The enzymes regulated by two‐component regulatory systems (e.g., PhoP/PhoQ and PmrA/PmrB) are responsible for these alterations, ultimately decreasing AMP affinity and enhancing bacterial survival.

### 5.2 Efflux Pump Expression

Efflux pumps are another critical mechanism contributing to AMP resistance and these membrane transporters actively expel AMPs from bacterial cells, preventing them from reaching their intracellular or membrane‐associated targets. A well‐documented example is the SbmA efflux transporter in *E. coli*, which confers resistance against proline‐rich AMPs by exporting these peptides from the cytoplasm.^[^ ^155^, ^156^ ^]^ Additionally, multidrug efflux pumps like MexAB‐OprM in *P. aeruginosa* ^[^ ^157^, ^158^ ^]^ and AcrAB‐TolC in *E. coli* ^[^ ^159^ ^]^ and *S. typhymurium* ^[^ ^160^ ^]^ also confer cross‐resistance to diverse antimicrobial compounds, including AMPs, antibiotics, and detergents and the increased expression of such efflux systems under AMP exposure highlights the adaptability of bacteria to various environmental stressors.

### 5.3 Protease‐Mediated AMP Degradation

Proteolytic degradation of AMPs by bacterial proteases represents a direct defensive strategy and certain pathogenic bacteria secrete proteases capable of cleaving AMPs before they can exert their activity. Some examples include the elastase LasB produced by *P. aeruginosa* ^[^ ^161^ ^]^ and proteases from *Porphyromonas gingivalis*.^[^ ^162^ ^]^ These enzymes degrade peptides extracellularly, significantly reducing their local concentration and efficacy and membrane‐bound proteases also degrade peptides after initial binding, further contributing to resistance. The susceptibility of AMPs to proteolytic degradation underscores the need for structural modifications such as incorporation of D‐amino acids, cyclization, or conjugation to polymers or lipids to enhance peptide stability and durability.^[^ ^163^, ^164^, ^165^ ^]^

### 5.4 Biofilm Formation and Extracellular Matrix Protection

Biofilms represent a complex and robust protective environment where bacteria exhibit increased resistance to antimicrobial agents, including AMPs.^[^ ^166^ ^]^ Biofilm‐associated cells are embedded within an extracellular polymeric substance (EPS) composed of polysaccharides, proteins, and nucleic acids, creating a physical barrier that restricts AMP penetration.^[^ ^167^ ^]^ Furthermore, biofilm bacteria often exhibit metabolic heterogeneity and reduced growth rates, which inherently decrease susceptibility to peptides designed to disrupt actively metabolizing cells. Studies have consistently reported lower AMP or polymyxins efficacy against biofilm‐forming bacteria such as *P. aeruginosa*, *Klebsiella pneumoniae*, and *S. aureus*, highlighting the substantial challenge posed by biofilm‐related resistance.^[^ ^168^, ^169^ ^]^ Despite current reports of resistance to AMPs, several studies continue to support their potential in inhibiting biofilm formation or targeting the ECM.^[^ ^170^ ^]^ This potential can be further enhanced through the use of nanotechnology‐based strategies.^[^ ^171^ ^]^

### 5.5 Release of Outer Membrane Vesicles

Gram‐negative bacteria have evolved an additional defense mechanism through the production of outer membrane vesicles (OMVs) and these vesicles can sequester AMPs, effectively neutralizing their activity before they reach bacterial membranes.^[^ ^172^ ^]^ Studies have demonstrated this phenomenon in pathogens such as *Neisseria meningitidis*, *Helicobacter pylori*, and *P. aeruginosa*, where OMVs significantly decrease the concentration of active peptides in the local environment. The ability of OMVs to adsorb AMPs and prevent their interaction with bacterial cells provides a potent protective mechanism that complicates peptide‐based therapeutic interventions.^[^ ^173^ ^]^

### 5.6 Activation of Stress Response Pathways

Bacteria exposed to AMPs activate numerous stress‐response systems that facilitate adaptive resistance. Two‐component regulatory systems, such as CpxRA and σE envelope stress responses are prominently involved in detecting membrane disruption caused by AMPs and activating gene expression to reinforce bacterial defenses.^[^ ^174^, ^175^ ^]^ Activation of these systems leads to increased expression of efflux pumps, modification of outer membrane proteins, and reinforcement of membrane integrity and these adaptive responses enable bacteria to tolerate sublethal concentrations of AMPs, allowing them to survive, adapt, and develop stable resistance phenotypes.^[^ ^176^, ^177^ ^]^

### 5.7 Genetic and Adaptive Resistance

Bacterial populations continuously evolve under antimicrobial pressure, and prolonged or repeated exposure to AMPs can select genetically stable resistance mutations.^[^ ^14^, ^178^ ^]^ Laboratory evolution experiments have demonstrated that bacteria such as *S. aureus*, *P. aeruginosa*, and *E. coli* can acquire stable resistance mutations affecting genes associated with membrane biosynthesis, efflux pump expression, or AMP‐target interactions.^[^ ^179^ ^]^ Although genetic resistance generally arises at a slower rate compared to conventional antibiotics, the risk remains significant under sustained exposure. Furthermore, horizontal gene transfer mechanisms such as conjugation, transformation, and transduction facilitate the dissemination of resistance determinants within bacterial communities, increasing the potential risk of widespread AMP resistance.^[^ ^180^, ^181^ ^]^ To overcome the inherent limitations associated with antimicrobial peptides, innovative approaches have emerged to enhance their bioavailability and stability within the GIT. Among these innovative approaches, nanotechnological strategies are particularly notable due to their versatility and effectiveness as AMP delivery vehicles.^[^ ^182^ ^]^

## 6 AMP‐Loaded Nanocarriers as Drug Delivery Systems

AMPs represent a promising class of therapeutic agents. However, their clinical translation is hindered by limited stability and rapid degradation under physiological conditions, as discussed in Section 5.^[^ ^183^ ^]^ To overcome these barriers, advanced strategies involving peptide encapsulation and surface functionalization via nanoparticles have been developed to enhance peptide protection and bioavailability. These approaches extend peptide half‐life, mitigate off‐target effects, and may even enhance antimicrobial efficacy.^[^ ^184^ ^]^

Encapsulation involves embedding peptides within nanostructured matrices or coatings, typically composed of lipids, polymers, or inorganic materials, allowing improved transport and controlled release at target sites. Material selection is tailored according to peptide physicochemical properties and the intended therapeutic application.^[^ ^184^, ^185^ ^]^

Moreover, functionalization broadly refers to chemical modifications of the nanoparticle surface intended to impart desirable characteristics such as improved stability, increased cellular specificity, or enhanced permeability through biological barriers. A specific form of functionalization is conjugation, which explicitly involves anchoring bioactive molecules, such as peptides, to the nanoparticle surface.^[^ ^186^ ^]^ Conjugation enhances selective targeting, optimizes interactions with specific cellular receptors, and improves therapeutic efficacy by reducing systemic toxicity.^[^ ^187^, ^188^ ^]^ Peptide conjugation methods can be categorized into covalent and non‐covalent strategies. Covalent conjugation includes techniques such as EDC/NHS coupling (amine–carboxyl groups), thiol–maleimide chemistry, and bioorthogonal “click” reactions like azide–alkyne cycloaddition.^[^ ^187^, ^188^, ^189^ ^]^ Non‐covalent conjugation relies on reversible electrostatic or hydrophobic interactions, enabling peptide adsorption onto nanoparticle surfaces without direct chemical linkage.^[^ ^190^, ^191^ ^]^ Selection between these methods depends on the desired stability, peptide properties, nanoparticle material, and intended biomedical application.

This section provides a comparative overview of the most relevant nanocarrier systems, such as solid lipid nanoparticles, nanostructured lipid carriers, liposomes, polymeric nanoparticles, metallic nanoparticles, and mesoporous silica nanoparticles (**Table** **3**), highlighting recent advances and applications of AMP‐loaded and peptide‐conjugated nanoparticles for the treatment of gastrointestinal infections (**Table** **4**).

**Table 3:** Comparative overview of nanocarriers for the delivery of antimicrobial peptides.

Nano Carrier	Composition (CP)	Loading capacity (LC)	Scalability	Toxicity	Advantages	Disadvantages	Refs.
SLNs	Solid lipid core (e.g., triglycerides, waxes), stabilized by surfactants	Moderate (limited by crystalline matrix)	Industrial methods (high‐pressure homogenization, microemulsion) are established	Generally safe lipid components; surfactant choice can cause irritation	‐Protects peptides from gastrointestinal conditions. ‐ Good physical stability	‐Drug expulsion over time. ‐ Polymorphic transitions may reduce loading and stability	[192, 193]
NLCs	Blend of solid and liquid lipids, resulting in a less ordered lipid matrix	Higher than SLNs	Similar to SLNs; relatively straightforward to scale up	Usually biocompatible; attention to surfactant concentration needed	‐Greater loading capacity vs SLNs. ‐ Less risk of drug leakage. ‐ Less risk of drug leakage. ‐ Prolonged reléase.	‐Requires balanced lipid ratios. ‐ Susceptible to lipid oxidation if not well protected	[192, 193]
LPS	One or more phospholipid bilayers (optionally containing cholesterol)	Moderate to high (depending on lipids)	Commercially viable; liposomal drug formulations are already on the market	Low toxicity due to phospholipids; sometimes cleared rapidly by the immune system	‐ Can carry both hydrophilic (aqueous core) and lipophilic (bilayer) molecules	‐Potentially short shelf life. ‐ High costs for phospholipids. ‐ Possible leakage/fusion	[194, 195]
PNP	Natural or synthetic polymers (e.g., PLGA, chitosan, alginate)	Often high (engineered designs)	Various established methods; PLGA systems already FDA‐approved	Generally safe for biodegradable polymers; cationic polymers can show cytotoxicity	‐Tunable release profiles. ‐ Typically stable in storage. ‐ Versatile materials	‐Possible use of organic solvents. ‐ Complex formulation processes. ‐ Cost can increase	[196, 197]
MNPs	Metal cores (gold, silver, iron oxide), often surface‐functionalized	Moderate (mainly surface‐based loading)	More complex; requires precise control of size, shape, and surface chemistry	Potential toxicity from ion release (especially silver); gold is more inert	‐Strong antimicrobial effect (e.g., silver ions), ‐ Easy to functionalize surface	‐Risk of bioaccumulation/toxicity. ‐ Environmental concerns. ‐ May require high‐quality control	[198, 199]
MSNs	Silica matrix with tunable pores (2–50 nm), large surface area and pore volume	Moderate (depends on the pore size)	Can be scaled via sol‐gel and templating methods; requires strict pore‐size control	Generally biocompatible; poorly designed particles may induce inflammation	‐Moderate loading capacity. ‐ Stable and customizable pore structure. ‐ Controlled release	‐There is a risk of accumulation in the body if they do not degrade properly. ‐ Surface modifications crucial for biocompatibility	[200, 201]

CP: Composition; LC: Loading Capacity, SLNs: Solid Lipid Nanoparticles, NLCs: Nanostructured Lipid Carriers, LPS: Liposome, PNP: Polymeric Nanoparticles, MNPs: Metallic Nanoparticles, MSNs: Mesoporous Silica nanoparticles

**Table 4:** In vivo applications of peptide‐loaded and peptide‐conjugated nanoparticles against gastrointestinal infections.

Nanocarriers	Size (nm)	In vitro effect	Dose and animal model	In vivo effect	Zeta potential (mV)	Refs.
Omi‐hyd‐Dex@HA Nanoparticles	96.6	The Omi‐hyd‐Dex@HA nanoparticles exhibited a broad spectrum against *S. aureus*, *P. aeruginosa*, *Klebsiella pneumoniae* (KP), and *Candida albicans*, and, similar to omiganan, increased bacterial membrane permeability.	Intravenous injection at a dose of 8.0 mg kg^−1^ in murine models.	The Omi‐hyd‐Dex@HA nanoparticles significantly increased survival rates compared to clinical antibiotic combinations.	−32.7	[258]
AgNP@AMP/SF	20	The antibacterial test against *S. aureus* showed an inhibition rate >99%, due to cell membrane disruption and the generation of reactive oxygen species (ROS).	Male Sprague–Dawley (SD) rats via insertion of a titanium rod.	The SF coating improved adhesion, diffusion, and proliferation of BMSCs, promoting osteogenic gene expression, collagen secretion, and osteointegration at weeks 4 and 8.	N/A	[249]
CNM	295.3 ± 96.4	CNM exhibited high antibacterial activity against *E. coli* O157:H7, *E. coli* K88, and methicillin‐resistant *S. aureus*, damaging their cell membranes and maintaining stability against temperature and pH variations, unlike the narrow‐spectrum MccJ25.	CNM and chitosan at 0.025%, 0.05%, and 0.1% administered by feeding in *C. elegans*.	Treatments with 0.05% and 0.1% CNM reduced the lifespan of *C. elegans*, whereas worms treated with 0.025% CNM did not show toxicity.	N/A	[233]
AGO‐AgNPs‐OA	100	AGO‐AgNPs and AGO‐AgNPs‐OA showed greater antibacterial effect against gram‐negative bacteria, with zones of inhibition of 20 ± 1 mm and 22.3 ± 1.2 mm, respectively.	Male Sprague–Dawley rats (300–350 g) with induced wounds, with dressings applied every three days.	AGO‐AgNPs‐OA accelerated wound healing, reduced scarring, improved inflammation, collagen deposition, and angiogenesis.	−23.42 ± 0.19	[259]
MSI‐1/PB@CS/PVA Mixed with PBNP	115	MSI‐1/PB@CS/PVA + NIR, with PBNP, eliminated *S. aureus* and *E. coli* in 24 h (p < 0.01). It showed 90% cell viability and 0.35% hemolysis.	Male BALB/c mice with wounds infected with *E. coli* and *S. aureus* (10 µL, 1×10⁸ CFU mL^−1^), treated with hydrogels 24 h after infection.	MSI‐1/PB@CS/PVA + NIR eliminated *S. aureus* and *E. coli* in vivo in 3 days (p < 0.01, 100% reduction), whereas other hydrogels showed a sustained effect until day 7. PBNP only enhanced the activity with irradiation.	N/A	[260]
SF@CBF‐EGCG	191.7 ± 4.3	Reduced ROS (≈100%) and RNS (≈90.3%), decreased TNF‐α and IL‐12 while increasing IL‐10, inhibited *E. coli* (≈90%) and *S. aureus* (>20%), and effectively adsorbed LPS.	Female BALB/c mice, administered orally with nanoparticles embedded in a hydrogel (5.0 mg EGCG kg^−1^ or 166.7 mg blank nanoparticles kg^−1^).	Exhibited a significant therapeutic effect in mice with ulcerative colitis, reducing body weight loss by 4.6%, improving colon length and epithelial barrier protection, with a notable 60% reduction in inflammatory cytokines (TNF‐α and IL‐12).	−24.1 ± 0.7	[261]
AuNR@C‐At5	AuNR@C‐At5: 50;	Complete bactericidal effect against *E. coli* at 15 µg mL^−1^ and against *S. aureus* at 5–10 µg mL^−1^, with significant improvement when applying laser irradiation—especially at 25 µg mL^−1^.	Male BALB/c mice injected with *S. aureus* (10⁷–10⁸ CFU mL^−1^) and treated with 6.75 µg mL^−1^ C‐At5, 15 µg mL^−1^ AuNR@C‐At5, AuNR@PEG with laser, and AuNR@C‐At5 with laser (1 W/cm^2^, 30 s).	AuNR@C‐At5 accelerated wound healing and nearly completely eliminated residual bacteria by day 12, showing greater angiogenesis and lower inflammation than AuNR@PEG.	+29.9	[262]
PLGA‐pTA‐Der	345 ± 0.7 nm	Potent synergistic antibacterial activity against *S. aureus* and *E. coli*, with a 99% elimination rate after NIR irradiation. This treatment reduced the Young's modulus from 40,576 ± 1,130 MPa to 4,161 ± 0.323 MPa in *S. aureus* and from 682,572 ± 7,554 MPa to 37,916 ± 0.288 MPa in *E. coli*, indicating severe structural damage to the bacterial membrane.	BALB/c mice (n = 7 per group) with wounds infected with 50 µL of *S. aureus* (1×10⁸ CFU mL^−1^) were treated with PLGA‐pTA (16 µg mL^−1^) and Der (8 µg mL^−1^), with or without NIR laser irradiation (808 nm, 2.0 W/cm^2^, 5 min).	Accelerated healing of infected wounds, achieving 100% closure in 11 days. The wound temperature increased to 43.1 °C with irradiation, improving tissue regeneration, reducing inflammation (TNF‐α), and promoting angiogenesis (CD31).	−43.8 ± 0.39	[263]
LL‐37@MIL‐101‐Van	≈320 nm	Exhibited antibacterial activity against *S. aureus* (MIC: 4 µg mL^−1^), MRSA (MIC: 2 µg mL^−1^) and *E. coli*, with a synergistic effect mediated by •OH generation. Also showed high biocompatibility (hemolysis: 5.94% at 200 µg mL^−1^) and low cytotoxicity.	KM mice injected with 1.0×10⁸ CFU of methicillin‐resistant *S. aureus* (100 µL) and, after 48 h, treated with PBS, Van (20 µg mL^−1^), MIL‐101 (100 µg mL^−1^), MIL‐101‐Van (100 µg mL^−1^), LL‐37 (10 µg mL^−1^), and LL‐37@MIL‐101‐Van (100 µg mL^−1^).	Significant improvement in wound healing, with a 92.48% reduction in wound area in 12 days compared to at least 70% in control groups. Additionally, it showed good bacterial targeting and low toxicity, with no changes in body weight or organ histology.	16.8 ± 1.1	[264]
PLGA and Polymyxin B Nanocapsules	92 ± 25 nm	The MIC was evaluated in *E. coli* (4 µg mL^−1^) and *P. aeruginosa* (8 µg mL^−1^), showing a reduction in biomass and CFU. Cytotoxicity in renal cells was low according to the MTT assay.	*Galleria mellonella* larvae at 11.8 µg mL^−1^.	Showed significant antimicrobial efficacy, achieving 100% survival in larvae infected with *E. coli*, whereas polymyxin B and PLGA alone reached only 30%. Additionally, no toxic effects were observed, indicating greater safety and efficacy of the nanocarrier compared to controls.	−0.15 ± 0.04	[265]
Ura56‐PEG‐AuNP	∼10	Ura56‐PEG‐AuNP exhibited in vitro bactericidal activity with an MIC of 0.13 µM for *S. aureus* and 0.25 µM for *E. coli*. Its mechanism involves membrane disruption, alteration of zeta potential, and ROS generation.	Study in mice with doses of 187.5 µM and 375 µM of Ura56‐PEG‐AuNP.	In mice, Ura56‐PEG‐AuNP at 375 µM caused subcutaneous accumulation and a red coloration. At 187.5 µM and 375 µM, mild inflammation was observed. No toxicity was observed at 100 µM or lower. Coculture showed bactericidal selectivity without damaging mammalian cells.	58.5	[266]
HSN@RL‐QN15/ZA	∼320	The HSN@RL‐QN15/ZA hydrogel demonstrated antibacterial activity against bacteria, fungi, and MRSA through the release of zinc ions. The individual components did not show activity. The MIC for *S. aureus* was 32 µg mL^−1^.	Induced bleeding in mice followed by topical application.	In MRSA‐infected mice, HSN@RL‐QN15/ZA accelerated wound healing, with a high contraction rate (94.56% at 9 days) and improved re‐epithelialization, vascular regeneration, and collagen formation.	N/A	[267]
GAAP	25 ± 2.2	GAAP showed in vitro antibacterial effect against *Staphylococcus aureus* with an MIC of 5 µg mL^−1^.	Applied on rat skin at a dose of 10 µg mL^−1^.	GAAP eliminated *S. aureus* (99.99% in 24 h, 99.999% in 48 h), prevented biofilm formation in 4 h, and restored tissue without inflammation or necrosis.	16.1 ± 2.7	[268]
SPDN	60–140	C16‐2RP and C16‐3RP have MICs of 2.94 and 2.16 µM against *E. coli* and other bacteria. They induce membrane disruption and cell death and are stable in serum, with low hemolysis and cytotoxicity.	Intraperitoneal injection of 10, 20, or 40 mg kg^−1^ in mice.	C16‐3RP nanoparticles reduced bacterial load and pro‐inflammatory cytokines (P < 0.001) in mice infected with *E. coli* ATCC25922, improving tissue lesions.	N/A	[269]
CS@AMP‐NRC07	153 ± 35	CS@AMP‐NRC07 reduced *P. aeruginosa* (MIC: 0.7–45 µM) and were more cytotoxic against MCF‐7 and MDA‐MB‐231 than the free peptide. Empty CS‐NP also exhibited antitumor effects.	Immunodeficient mice.	Triggers an immune response and causes oxidative stress	+40.6 ± 2.0	[270]
Oct@CS	372.80 ± 2.31	Oct@CS exhibited a higher MIC and maintained 97.83% cell viability at 400 µg mL^−1^, superior to the 85.19% observed with free octominin.	Zebrafish larvae (0–100 µg mL^−1^).	Oct@CS did not cause mortality or abnormalities up to 50 µg mL^−1^, whereas free octominin caused 100% mortality at 50 and 100 µg mL^−1^.	+51.23 ± 0.38	[271]
PCM	298,5	PCM NPs increased cell viability from 39% to 78% at 400 µg mL^−1^, reduced ROS and pro‐inflammatory cytokines (IL‐6, IL‐1β, TNF‐α), and increased IL‐10, in addition to exhibiting enzyme‐mimetic activity (SOD, GPx, and POD).	Male C57BL/6 mice received 150 µL of PCM NPs (1 mg mL^−1^ PDA NPs + 1 mg mL^−1^ mCRAMP) by oral gavage.	PCM NPs alleviated DSS‐induced colitis by reducing the disease activity index (DAI) and restoring the intestinal barrier, increasing Bacteroidetes and Firmicutes, and reducing Akkermansia and Alistipes.	−37,4 ± 3,6	[272]
NapFab@DMSN	163 ± 7	In human macrophages infected with *Mycobacterium tuberculosis*, NapFab@DMSN at 34 µg mL^−1^ reduced the intracellular growth of *M. tuberculosis* by 80%.	Zebrafish embryos (*Danio rerio*) exposed to NapFab@DMSN at 34 µg mL^−1^ for 24 h starting from 24 h postfertilization.	It did not cause significant mortality or toxicity in zebrafish embryos, with no effects on development, heart, or the nervous system (Chi‐Square, p > 0.05).	−17.5 ± 0.4	[273]
PhaNP@Syn71	20.2 ± 2.4	Inhibited the growth of *S. pyogenes* by more than 99.99% in a dose‐dependent manner and exhibited minimal cytotoxicity in human HaCaT keratinocytes up to 64 µM.	C57BL/6 mice with wounds infected by *Streptococcus pyogenes*, treated with a topical dose of 150 µM and an intravenous dose of 100 µM of PhaNP@Syn71.	Promoted infection‐free wound healing in mice by stabilizing wound size immediately after application and demonstrating high biocompatibility.	−8.54	[274]

*AgNP@AMP/SF: Silver Nanoparticles with AMP and Bionic Fibroin, CNN: Chitosan Nanoparticles with Antimicrobial Peptide Microcin J25, AGO‐AgNP‐OA: Agarose Oligosaccharides with Silver Nanoparticles and Odorranain A, PBNP: Prussian Blue Nanoparticles, CS: Chitosan, MSI‐1/PB@CS/PVA: Chitosan‒Polyvinyl Alcohol Nanoparticles Functionalized with the MSI‐1 Peptide, SF@CBF‒EGCG: Silk Fibroin Nanoparticles Loaded with Epigallocatechin‐3‒Gallate and Functionalized with the CBF Peptide, AuNR@C‒At5: Gold Nanorods Modified with Peptide C‒At5, PLGA‒pTA‒Der: PLGA Nanoparticles Coated with Polytannic Acid and Decorated with Dermaseptin‐PP, LL‐37@MIL‐101‐Van: Material of Institute Lavoisier 101 Nanoparticles Functionalized with LL‐37LL‒37 Peptide and Vancomycin, Ura56‐PEG‐AuNP: Gold Nanoparticles Functionalized with Lipopeptide Ura56, HSN@RL‐QN15/ZA: Zn^2^⁺‐Crosslinked Alginate Containing Hollow Silica Nanoparticles Loaded with RL‐QN15 Peptides, GAAP: Graphene‐Silver Nanocomposites Functionalized with Antimicrobial Peptides, SPDN: Self‐Assembling Dendritic Peptide Nanoparticles, CS@AMP‐NRC07: NRC07 Peptide Loaded in Chitosan Nanoparticles, Oct@CS: Octominin Peptide Loaded in Chitosan Nanoparticles, PCM: Polydopamine Nanoparticles Loaded with the mCRAMP Peptide and Coated with Macrophage Membranes, NapFab@DMSN: Dendritic Mesoporous Silica Nanoparticles Loaded with Napsin‐Derived Peptide, PhaNP@Syn71: Phage‐Mimicking Nanoparticles Conjugated with the Antimicrobial Peptide Syn71.

### 6.1 Solid Lipid Nanoparticles

Solid lipid nanoparticles (SLNs) have been increasingly used as promising drug delivery nanocarriers for various diseases, including microbial infections, diabetes, neurological disorders, skin diseases, and especially cancer.^[^ ^202^ ^]^ These nanoparticles are based on a crystalline lipid core stabilized by surfactants and, in some cases, by cosurfactants.^[^ ^203^ ^]^ The solid core composition of SLNs, which generally consists of fatty acids, fatty alcohols, waxes, and mono‐, di‐, and triglycerides, can affect their properties. SLNs are solid at physiological temperature, biocompatible, and biodegradable, and are capable of encapsulating both hydrophilic and lipophilic drugs, which can improve their stability, solubility, and bioavailability.^[^ ^204^, ^205^ ^]^

Despite their advantages, the application of SLNs as drug delivery nanocarriers faces several challenges. Physicochemical instabilities, such as recrystallization, polymorphic transitions, drug leakage during storage, and insufficient drug loading, limit the shelf life and clinical potential of these materials.^[^ ^206^, ^207^ ^]^ However, recent studies have demonstrated significant progress in overcoming these limitations.

**Figure 3:**
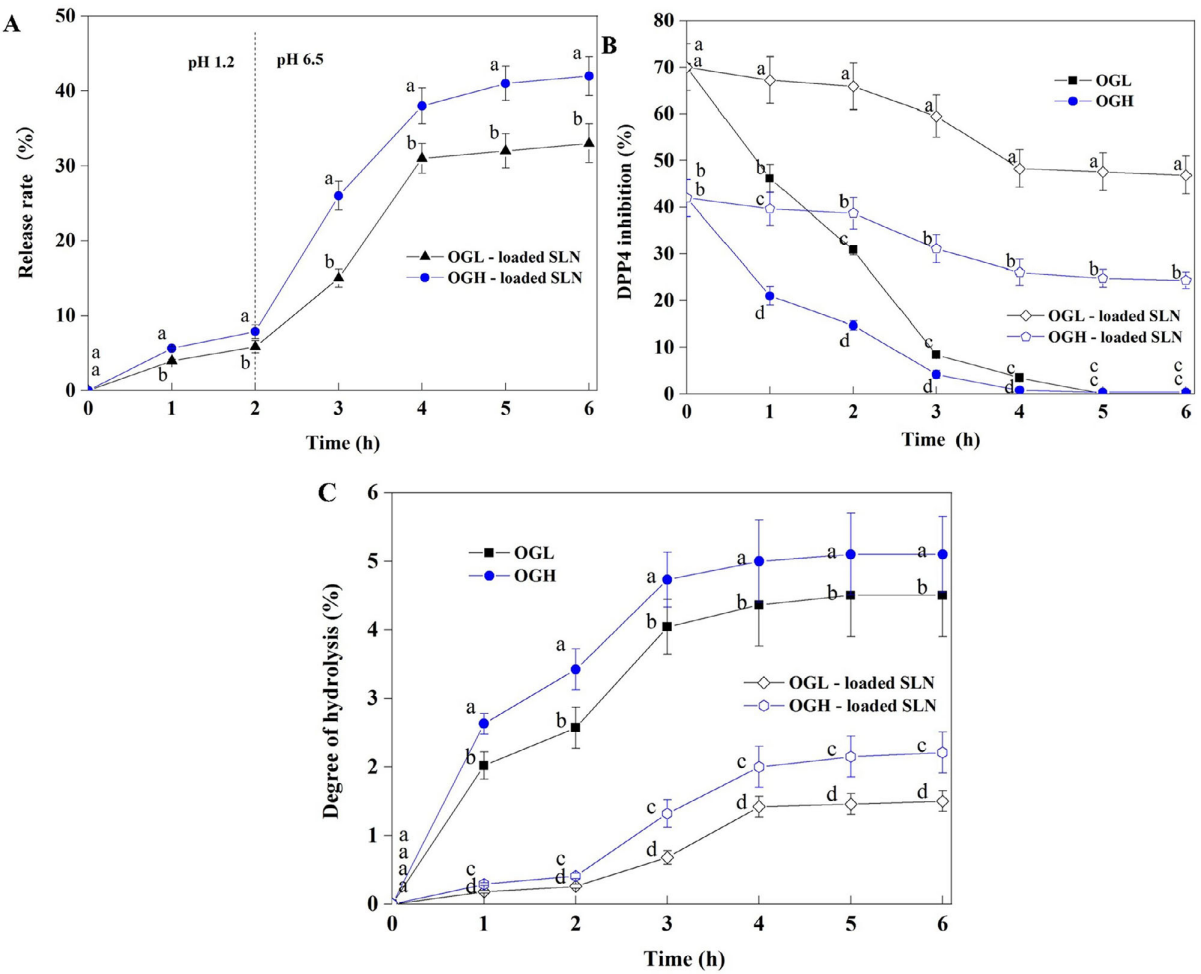
In vitro evaluation of the release, inhibition, and stability of globulin peptide fractions and their SLN formulations after simulated gastrointestinal digestion. A) Release rates (%) of OGL and OGH from SLN at pH values of 1.2 and 6.5. B) DPP4 inhibition (%) over time for OGL, OGH, and their SLN formulations. C) Degree of hydrolysis (%) of OGL and OGH compared with their encapsulated versions. Reproduced with permission.^[^ ^208^ ^]^ Copyright 2020, Elsevier B.V.

Su et al.^[^ ^208^ ^]^ investigated the use of SLNs as carriers to preserve the structural integrity of peptides under adverse GIT conditions. In this study, two fractions of peptides derived from oat globulin were encapsulated, resulting in nanoparticles with specific characteristics depending on the type of peptide. SLNs containing low‐molecular‐weight (OGL) peptides (<3 kDa) had an average diameter of 163.9 nm and a polydispersity index of 0.21, whereas those containing moderate‐molecular‐weight (OGH) peptides (3–10 kDa) had an average size of 270.9 nm and a polydispersity index of 0.44. Both formulations exhibited colloidal stability, with zeta potentials of approximately −20 mV. In simulated gastrointestinal fluids, the SLNs provided controlled peptide release, which was slower for particles with low‐molecular‐weight peptides. The latter achieved a maximum release of approximately 7.87% in gastric fluid and 42% in intestinal fluid after 6 h (**Figure** **3**A). The nanoparticles maintained high dipeptidyl peptidase IV (DPP4) inhibitory activity, preserving the functionality of the peptides and preventing their degradation. The nonencapsulated OGL fraction rapidly decreased DPP4 inhibition, decreasing from 70% to 30.8% and 8.4% at 0, 2, and 3 h of incubation, respectively, whereas the OGH fraction decreased from 42% to 14.7% and 4.2%, respectively, at the same time points. In contrast, SLN‐loaded peptides remained significantly more stable, with DPP4 inhibitory rates of 70%, 65.9%, and 48.3% for SLN‐OGL at 0, 2, and 4 h, respectively, and 42%, 38.69%, and 26.04% for SLN‐OGH (Figure 3B). Furthermore, hydrolysis analysis revealed that nonencapsulated peptide fractions underwent considerable enzymatic degradation in gastrointestinal fluids, with final hydrolysis degrees (DHs) of 3.6% for OGH and 3.1% for OGL. However, SLN encapsulation significantly reduced peptide hydrolysis, with final DH values of only 0.86% and 0.6% for SLN‐OGH and SLN‐OGL, respectively, demonstrating the protective effect of SLN against enzymatic degradation (Figure 3C). These results demonstrate the effectiveness of SLNs as systems for the protection and delivery of bioactive peptides in oral applications.

Similarly, Ryan et al.^[^ ^209^ ^]^ demonstrated that SLNs significantly enhanced the antimicrobial activity of the bacteriocin lacticin 3147 against *Listeria monocytogenes*. The SLNs had a particle size of ≈85 ± 3 nm and a zeta potential of −10.2 mV. After the peptides Ltnα and Ltnβ were encapsulated, an increase in zeta potential was observed, reaching +4.6 mV for Ltnα and +3.6 mV for Ltnβ, indicating interaction with the nanoparticle surface. In terms of activity, the encapsulated SLNs had an inhibition zone of 17.6 ± 0.5 mm, whereas the free peptides had an inhibition zone of only 12.9 ± 0.3 mm, representing a 36.4% improvement in antimicrobial activity. Moreover, encapsulation protected the peptides from enzymatic degradation by α‐chymotrypsin, with inhibition zones of 7.4 ± 0.9 mm compared with 6.0 ± 0.1 mm for free peptides. This demonstrates greater stability and protection against enzymatic degradation, properties that are essential for in vivo applications.

Ratrey et al.^[^ ^210^ ^]^ encapsulated nisin Z in SLNs (SLNs@nisin). SLNs@nisin reduced the MIC of the free peptide from 40 µg mL^−1^ to only 10 µg mL^−1^ against *S. aureus*. Additionally, these nanoparticles, at a concentration of 10 µg mL^−1^ nisin Z, achieved a complete bactericidal effect in just 30 minutes, whereas the free peptide at the same concentration failed to completely eliminate the bacteria even after 120 min of exposure. The synthesized SLNs had an average size of 79.47 ± 2.01 nm and a zeta potential of −9.8 ± 0.3 mV, suggesting adequate stability for interaction with bacterial membranes. TEM and scanning electron microscopy (SEM) revealed that bacteria treated with SLNs presented larger and more numerous pores than those treated with the free peptide did, indicating greater membrane‐damaging capacity. Flow cytometry assays confirmed that bacterial membrane damage was significantly greater with SLNs encapsulating nisin Z. Notably, the SLNs showed no antimicrobial activity on their own, confirming that their effect depends entirely on synergy with the encapsulated peptide. In terms of safety, the SLNs demonstrated low toxicity toward human cells and did not cause significant hemolysis in human erythrocytes. These results indicate that encapsulating nisin Z in SLNs enhances its antimicrobial efficacy by improving membrane disruption and reducing the MIC while ensuring low cytotoxicity and hemolytic activity, thus offering a safer and more effective delivery system for therapeutic applications.

### 6.2 Nanostructured Lipid Carriers

Nanostructured lipid carriers (NLCs) represent a second generation of lipid nanoparticles designed to overcome the limitations of the first generation (limited drug‐loading capacity and the potential for drug expulsion during storage owing to the crystallization of the solid lipid matrix), namely, SLNs.^[^ ^211^ ^]^ Biodegradable and compatible lipids in the solid and liquid phases are used in their formulation. The solid lipids include oleic acid, caprylic triglycerides, and α‐tocopherol, whereas the liquid lipids include Copritol 888 ATO, Preciriol ATO5, and stearic acid.^[^ ^212^ ^]^ The incorporation of liquid lipids causes structural imperfections in solid lipids, leading to a less ordered crystalline arrangement that prevents drug leakage and provides a high payload capacity.^[^ ^213^ ^]^ A nanocarrier should possess properties such as solubility in biological environments, prolonged biological half‐life, biocompatibility, protection capabilities, ability to bind to target areas, and easy intracellular penetration.^[^ ^214^ ^]^

In recent years, NLCs have attracted attention as alternatives to SLNs, polymeric nanoparticles, emulsions, microparticles, liposomes, and others.^[^ ^182^ ^]^ These nanocarriers are suitable for delivering both hydrophilic and lipophilic drugs and are useful for oral, parenteral, ocular, pulmonary, topical, and transdermal pharmaceutical applications.^[^ ^192^, ^215^ ^]^ Among their advantages, they have a higher drug‐loading capacity than SLNs do and prevent drug expulsion during storage. However, they are associated with cytotoxic effects, and surfactants can cause irritation and sensitization.^[^ ^216^ ^]^

**Figure 4:**
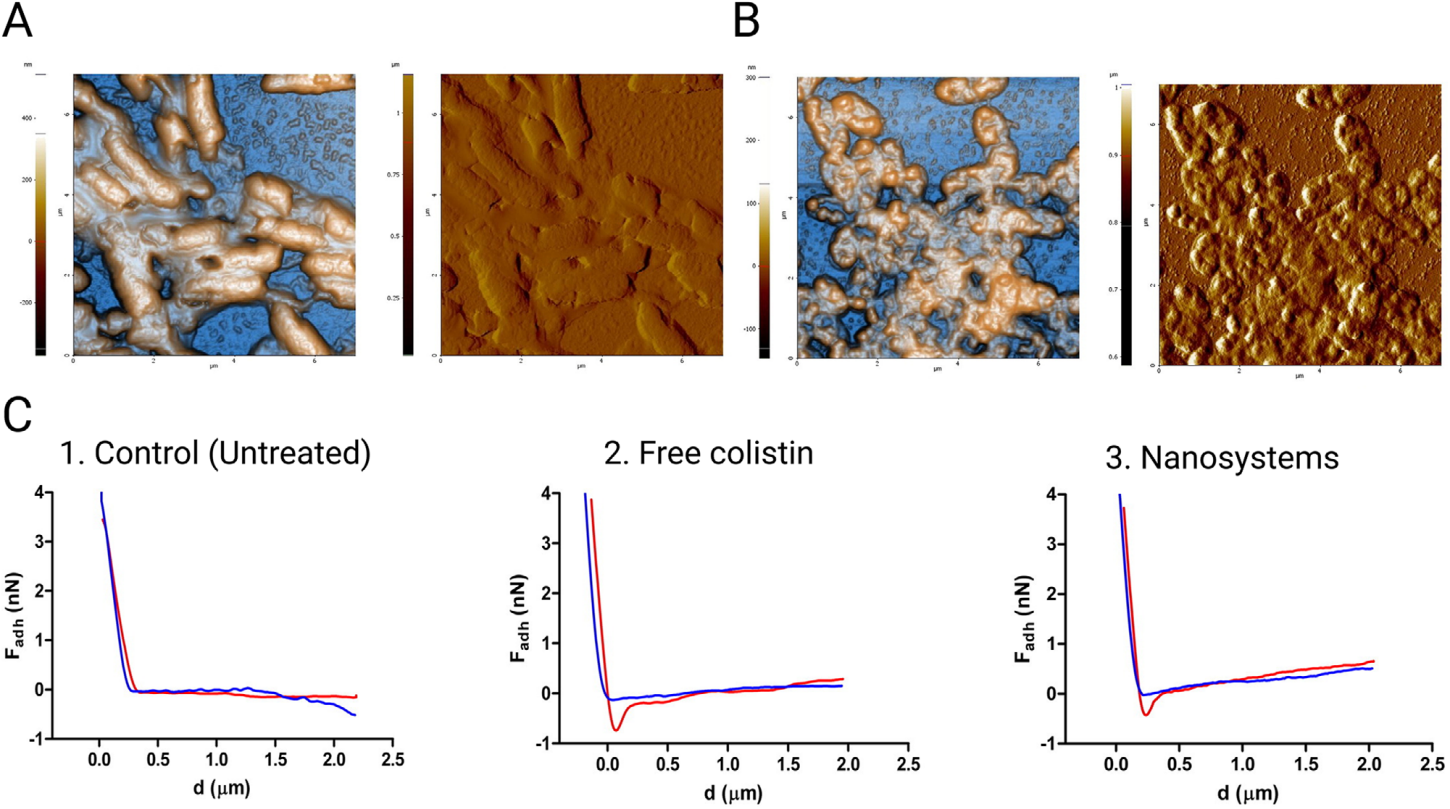
AFM images obtained at a 7 µm^2^ scan size of A) untreated P. aeruginosa biofilms; B) after treatment with 128 µg mL^−1^ free (first row) and nanoencapsulated (second row) colistin sulfate at 6 h. C) Typical force‒distance curves of P. aeruginosa untreated (1) and treated with colistin (2) and the nanocarrier (3). Reproduced with permission.^[^ ^218^ ^]^ Copyright 2016, Elsevier B.V.

Colistin is a peptide that interacts with lipopolysaccharides on the outer membrane of gram‐negative bacteria, primarily causing the displacement of calcium and magnesium ions. This mechanism significantly reduces outer membrane stability and increases permeability, leading to bacterial death by releasing the cytoplasmic content.^[^ ^217^ ^]^ In this context, Sans‐Serramitjana et al.^[^ ^218^ ^]^ encapsulated colistin in SLN and NLC to evaluate its efficacy against *P. aeruginosa*. The nanoparticles had an average size of 300–427 nm, with an encapsulation efficiency of 80–95%, ensuring sustained drug release, with at least 50% released within the first 24 h. Atomic force microscopy (AFM) revealed significant alterations in the structure of biofilms treated with nanoencapsulated colistin, increasing surface roughness (**Figure** **4**A,B). The measured adhesion force was 145 ± 33 nN in untreated cells, whereas it increased to 885 ± 195 nN with encapsulated colistin and 701 ± 239 nN with free colistin, indicating greater interaction with the bacterial membrane (Figure 4C). The antimicrobial activity against planktonic bacteria revealed that both free and nanoencapsulated colistin had MICs ≤ 4 µg mL^−1^ for most isolates. However, for biofilm eradication, free colistin required concentrations ranging from 640 to 2560 µg mL^−1^, whereas colistin encapsulated in NLCs successfully eliminated biofilms at only 160–320 µg mL^−1^. Additionally, in bacterial growth assays, 8 × MIC concentrations of free colistin (16 µg mL^−1^ for 056SJD and 256 µg mL^−1^ for P19) immediately inhibited bacterial growth, but nanoparticles further improved the efficacy against resistant strains. These results demonstrate that colistin encapsulation in lipid nanoparticles enhances antimicrobial and antibiofilm activity, making it an ideal strategy to combat resistant infections.

Sharaf et al.^[^ ^219^ ^]^ highlighted the advantages of using nanocarriers, specifically NLCs, for loading peptides such as hesperidin and clarithromycin in the treatment of *H. pylori*. The optimized NLCs had an average particle size of 19.6 nm and a negative Z‐potential of ‐19.4 mV, ensuring high system stability. This nanometric size enhances bioavailability by facilitating penetration into the bacterial membrane and protecting the encapsulated drugs from the acidic environment of the stomach. The NLCs achieved controlled and prolonged drug release over 24 h, with encapsulation efficiencies of 78% for hesperidin and 28% for clarithromycin, reducing the frequency of drug administration and improving antimicrobial effectiveness. In vitro studies demonstrated time‐ and concentration‐dependent inhibition of *H. pylori* growth, reaching 94% inhibition at a concentration of 25 µM. The combined action of the components causes damage to the bacterial membrane, increasing permeability to the antibiotic and promoting bacterial eradication. Furthermore, the system showed biocompatibility, with no significant cytotoxic effects on human cells at concentrations less than 50 µM. These findings suggest that the encapsulation of AMPs in NLCs reduces the required AMP dosage, decreases the frequency of administration, and enhances antimicrobial activity against the main pathogen responsible for gastritis.

Rocha et al.^[^ ^220^ ^]^ utilized NLCs for the delivery of AMPs in the treatment of bacterial infections. Compared with conventional antibiotic solutions, the developed system, which was coated with polymyxin B, demonstrated a two‐ to threefold increase in antimicrobial activity against *P. aeruginosa*. This improvement is attributed to its ability to enhance stability and provide controlled drug release. The average size of the nanoparticles ranged from 230.9 to 255.7 nm, with a polydispersity index suitable for ensuring uniformity. The zeta potential ranged from −2.06 to +3.45 mV, indicating an efficient coating that optimizes interactions with negatively charged biological surfaces, such as those of the eye. Antimicrobial results revealed a significant reduction in the MIC required against *P. aeruginosa* and *Bordetella bronchiseptica*. Furthermore, the system prolongs the residence time at the site of action, reduces the administration frequency, and minimizes side effects, thereby improving therapeutic efficacy and patient adherence in clinical applications.

### 6.3 Liposomes

Liposomes are self‐contained, spherical lipid bilayer nanocarriers widely used in medicine for drug delivery.^[^ ^221^ ^]^ They are composed of phospholipids and sometimes cholesterol, making them biodegradable, biocompatible, and exhibit low toxicity and immunogenicity.^[^ ^222^ ^]^ Selecting an appropriate lipid composition for AMP delivery is crucial, as it influences the physical properties of liposomes, including their encapsulation efficiency, loading capacity and stability.^[^ ^186^ ^]^ Phosphatidylethanolamines are less prone to forming lamellar phases and are often chosen for designing fusogenic liposomes that can fuse with target cell membranes. Moreover, anionic phospholipids, such as phosphatidylglycerols, are generally avoided in AMP liposome carriers, as many cationic AMPs exhibit membrane‐disruptive activity in the presence of anionic phospholipids.^[^ ^223^ ^]^ Despite their advantages, liposomes face some obstacles that limit their application. These include a short shelf life, instability, low encapsulation efficiency, rapid clearance by the reticuloendothelial system, cellular interactions, a tendency for adsorption, and membrane transfer.^[^ ^224^ ^]^

Cantor et al.^[^ ^225^ ^]^ demonstrated the advantages of nanocarriers for peptide delivery, especially in terms of antimicrobial activity. The encapsulation of peptides in polymer‐coated liposomes, such as Eudragit E‐100, significantly improved their stability and bioactivity. The particle sizes of the nanocarriers were approximately 235 nm in noncoated liposomes and nearly doubled upon peptide loading, whereas the zeta potential shifted from negative to positive values after coating, confirming effective surface modification (−18 mV to +11 mV). The antimicrobial efficacy of the encapsulated peptides was significantly improved. Encapsulation increased the activity of the peptide +2 against *Listeria monocytogenes* by up to 2083 times, reducing the MIC to 0.06 µM. In the presence of *E. coli*, the activity increased 12.5‐fold, achieving an MIC of 1.25 µM in the encapsulated systems. These findings highlight the potential of these nanocarriers to combat foodborne pathogens.

**Figure 5:**
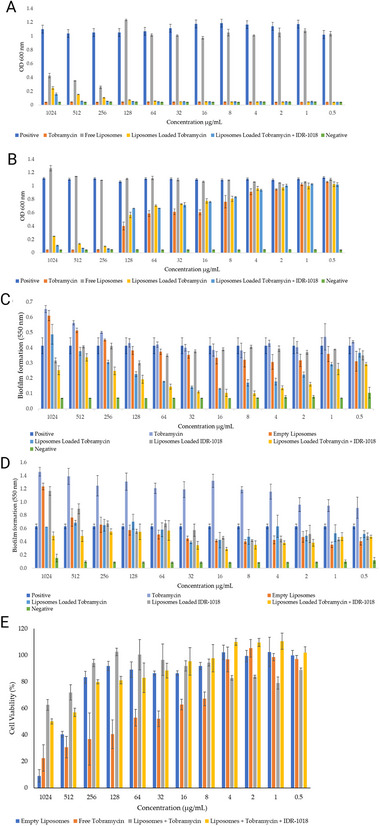
A) Effect of the applied formulations on PA01 (control strain), showing a significant reduction at all the tested tobramycin concentrations, while the MIC for free liposomes was 256 µg mL^−1^. B) Effect of the formulations on MDR 7067 (resistant strain), where both free and formulated tobramycin had an MIC of 256 µg mL^−1^, whereas empty liposomes showed no antibacterial activity. C,D) Biofilm formation in *P. aeruginosa* strains quantified by biomass measurements (OD 550 nm) after 48 h of exposure to free tobramycin and liposomal formulations: C) PA01 (control strain) and D) MDR 7067 (resistant strain). E) Viability of A549 cells after 48 h of exposure to different concentrations of empty liposomes, free tobramycin, tobramycin‐loaded liposomes, and tobramycin‐IDR‐1018‐loaded liposomes. Reproduced with permission.^[^ ^226^ ^]^ Copyright 2022, MDPI.

Alzahrani et al.^[^ ^226^ ^]^ encapsulated tobramycin and the IDR‐1018 peptide in liposomes to evaluate their efficacy against *P. aeruginosa* in reference (PA01) and multidrug‐resistant (MDR 7067) strains. The encapsulated tobramycin exhibited an encapsulation efficiency of 94 ± 2% and a particle size of less than 200 nm, with a zeta potential greater than 55 mV, suggesting colloidal stability and strong electrostatic interactions with the bacterial biofilm. MIC analysis revealed that tobramycin was the most effective antibiotic against PA01, with an MIC of ≤0.5 µg mL^−1^ in planktonic bacteria and 32 µg mL^−1^ in mature biofilms (**Figure** **5**A). For the MDR 7067 strain, the MIC was significantly greater (256 µg mL^−1^ in planktonic bacteria and >1024 µg mL^−1^ in biofilms), highlighting the high resistance of this strain (Figure 5B). Encapsulation of tobramycin in liposomes reduced biofilm formation in PA01 at concentrations between 4 and 32 µg mL^−1^ (p < 0.05), even without the IDR‐1018 peptide (Figure 5C). In the MDR 7067 strain, liposomes achieved a lesser reduction in biofilm formation, although the effect was more pronounced with IDR‐1018 (Figure 5D). With respect to cytotoxicity, tobramycin encapsulation improved its safety profile. While free tobramycin resulted in 37% cell viability at 256 µg mL^−1^, the encapsulated version increased viability to 94% at the same concentration in A549 lung cells (Figure 5E). The encapsulation of AMPs in liposomes optimizes antibiofilm activity and reduces cytotoxicity, two crucial factors in the treatment of resistant strains.

Faya et al.^[^ ^227^ ^]^ presented a formulation of AMPs whose activity was optimized against resistant bacteria, such as MRSA, through the use of pH‐sensitive liposomes. These systems offer multiple advantages: liposomes loaded with AMP‐2 and AMP‐3 (LP‐AMPs) have sizes of 102.6 ± 1.81 nm and 146.4 ± 1.90 nm, respectively, and zeta potentials of −9.81 ± 1.69 mV and −4.27 ± 1.25 mV at pH 7.4. At acidic pH (6.0), their size increases, and they exhibit a more positive charge, which promotes drug release. In antimicrobial tests, LP‐AMPs showed greater activity at acidic pH values than at neutral pH values, with AMP‐2‐Lipo‐1 demonstrating notable effectiveness, with an MIC of 1.95 µg mL^−1^ against MRSA. This design enhances cellular penetration and antibacterial activity, resulting in a reduction in the number of intracellular bacteria in infection models. Additionally, LP‐AMPs exhibited a sustained and safe release profile, with low toxicity and less than 1% hemolysis at effective concentrations. The results demonstrate that encapsulating AMPs in pH‐sensitive nanocarriers enhances their antimicrobial activity and broadens their therapeutic applicability against persistent infections.

### 6.4 Polymeric Nanocarriers

Polymeric nanocarriers (PNPs), which are composed of synthetic, semisynthetic, or natural polymers, have been extensively studied because of their ability to increase therapeutic efficacy and reduce the toxicity of pharmaceutical products.^[^ ^228^ ^]^ These polymeric nanoparticles offer the potential for a sustained release profile with unique pharmacokinetics for the effective delivery of drugs, AMPs, proteins, and DNA to specific organs or cells.^[^ ^229^ ^]^

The drug release rate depends on the characteristics of the polymer, preparation conditions, and properties of the polymer itself. Release mechanisms include diffusion, matrix erosion, or osmotic pressure.^[^ ^230^ ^]^ The preparation method determines how the therapeutic compound interacts within the polymer matrix through dissolution, binding, encapsulation, or entrapment. PNPs can be nanocapsules or nanospheres. Nanocapsules contain therapeutic compounds encapsulated within a polymer shell, whereas nanospheres contain compounds embedded in a polymer matrix.^[^ ^231^ ^]^

Different preparation methods for these nanoparticles include emulsification or interfacial polymerization, solvent displacement and interfacial deposition, solvent emulsification/evaporation, desolvation, and salting out.^[^ ^232^ ^]^ Yu et al.^[^ ^233^ ^]^ developed an antimicrobial nanocarrier based on chitosan nanoparticles (CNs) conjugated with the AMP microcin J25 (MccJ25), which has multiple advantages. This nanocarrier (CNM) had an average size of 295.3 ± 96.4 nm and showed a significant improvement in antimicrobial activity against both gram‐negative and gram‐positive bacteria, outperforming the individual components when used separately. CNMs were effective even at low concentrations (MIC of 0.05%) and exhibited stability under different temperature and pH conditions, ensuring their applicability in diverse environments. Moreover, the CNMs proved to be safe, showing no toxicity in cell lines or in an in vivo model of *Caenorhabditis elegans* at working concentrations. Importantly, they did not promote bacterial resistance or specific mutations after prolonged treatment. This system stands out for its synergistic action: CNs disrupt the bacterial cell membrane, allowing MccJ25 to inhibit RNA polymerase intracellularly, thereby increasing its effectiveness against multidrug‐resistant pathogens. In terms of antimicrobial activity, the CNMs demonstrated bactericidal capabilities against *E. coli* and MRSA, surpassing conventional antibiotics in terms of the spectrum and durability of action. These findings suggest that CNMs are promising candidates for combating antibiotic‐resistant infections, with the additional benefits of being less toxic and safer for clinical use.

**Figure 6:**
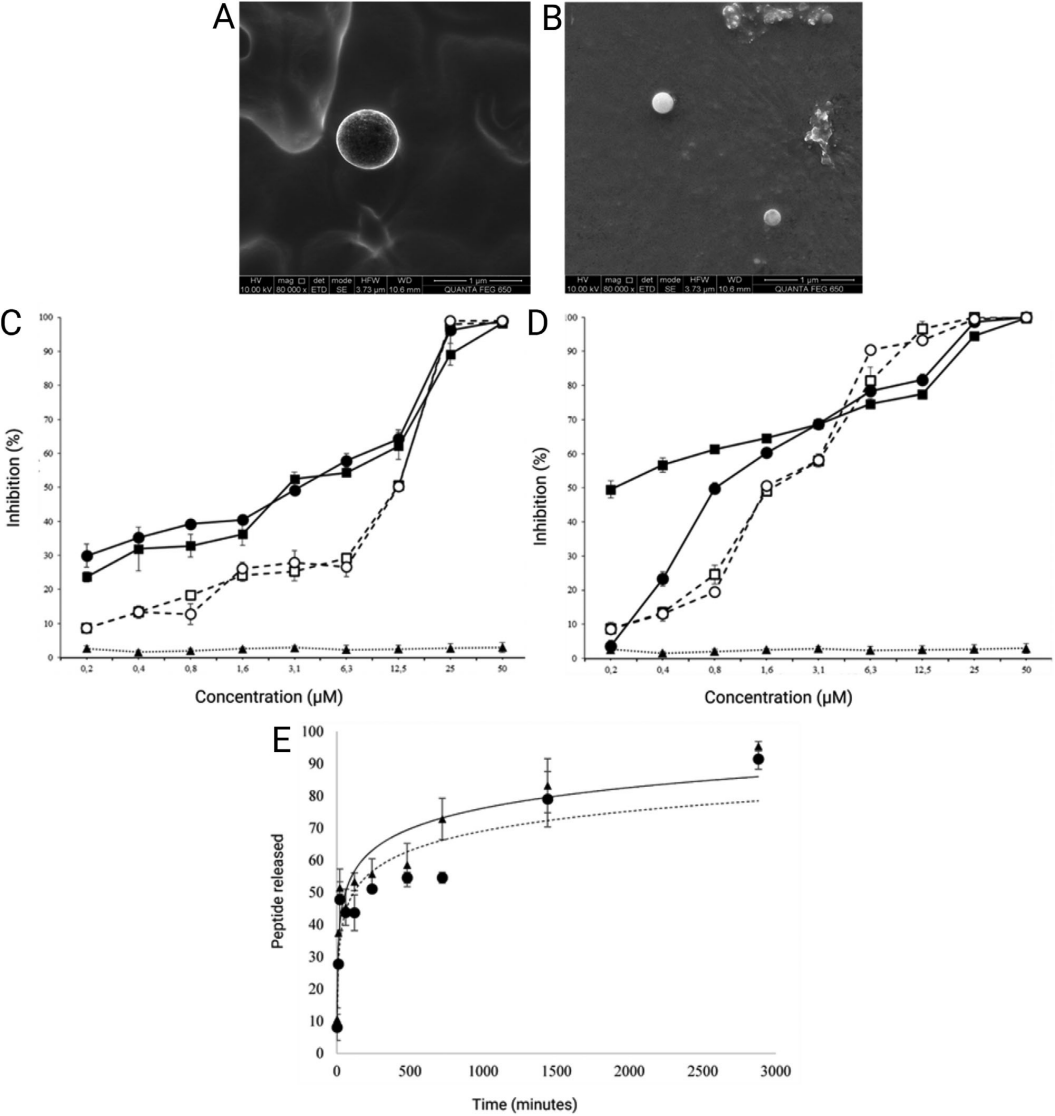
A) Micrographs of G17NP and B) G19NP obtained by scanning electron microscopy. (C) Antimicrobial activity of G19NP, G19, and NP against *E. coli* and D) MRSA. (■) G17NP, (□) G17, (▲) Empty nanoparticle, (●) G19NP, (○) G19. E) In vitro release profile of G17NP (▲) and G19NP (●) from polymeric nanoparticles under the same incubation conditions: 37 °C, pH 7.4, and 50 rpm. Reproduced with permission.^[^ ^234^ ^]^ Copyright 2020, MDPI.

Similarly, Gómez‐Sequeda et al.^[^ ^234^ ^]^ encapsulated the AMPs G17 and G19 in poly(lactic‐co‐glycolic acid) (PLGA) nanoparticles to increase their stability and antimicrobial activity against *E. coli* O157:H7 and MRSA. Encapsulation enabled an AMP loading of approximately 7 µg peptide/mg PLGA with an efficiency of 90.5%. The resulting nanoparticles had an average size of 290 nm (**Figure** **6**A,B) and a positive surface charge, facilitating interactions with bacterial membranes. Encapsulation reduced the MIC_50_ of G17 from 1.5 µM to 0.2 µM (G17NP) against MRSA and from 12.5 µM to 3.13 µM against *E. coli* O157:H7. For G19, the MIC50 decreased from 1.5 µM to 0.7 µM against MRSA and from 12.5 µM to 3.13 µM against *E. coli* O157:H7 (Figure 6C,D). Additionally, 44.0 ± 1.1% of G17 and 45.0 ± 1.1% of G19 were released within the first 60 min, followed by controlled release over 2880 min (Figure 6E). Toxicity tests revealed that the nanoparticles were neither cytotoxic nor hemolytic at effective concentrations. The empty nanoparticles exhibited no antimicrobial activity, confirming that the observed effect depended exclusively on the encapsulated peptide. These findings indicate that PLGA nanoencapsulation enhances the stability and efficacy of antimicrobial peptides, offering a promising option for combating resistant bacterial infections.

Casciaro et al.^[^ ^235^ ^]^ demonstrated that nanocarriers based on poly(lactic‐co‐glycolic acid) (PLGA) nanoparticles and the AMP esculentin (PLGA@ESCs) presented physical and functional properties that optimized their therapeutic efficacy. The nanoparticles had a hydrodynamic diameter of 261 to 282 nm and a near‐neutral zeta potential (‐0.67 to ‐0.84 mV), which facilitated their effective diffusion through biological barriers such as mucus and bacterial extracellular matrices without adverse interactions or aggregation. Additionally, these particles exhibited a biphasic release profile, with rapid initial release (≈60% within 3 h) followed by sustained release over three days, prolonging the antimicrobial activity compared with that of free peptides. In *P. aeruginosa* infection models, the intratracheal administration of PLGA@ESCs achieved a 3‐logarithm reduction in bacterial burden, which was significantly greater than that obtained with nonencapsulated peptides. This enhanced efficacy was attributed to the protection against degradation and the controlled release of the encapsulated AMPs. In in vitro studies, the nanoparticles maintained 60% inhibition of bacterial growth for 72 h, in contrast to the progressive decline in efficacy observed with free peptides over the same period. Moreover, biocompatibility studies revealed that these nanoparticles did not induce inflammatory responses in healthy mice, supporting their safety. These results highlight the potential of PLGA nanoparticles to improve the therapeutic efficacy and safety of AMPs against infections both in vitro and in vivo.

Moreover, the use of polymeric coatings in drug and bioactive delivery systems has become a widely adopted strategy in both the pharmaceutical and food industries.^[^ ^236^, ^237^, ^238^ ^]^ These coatings serve as protective barriers against gastrointestinal conditions, particularly gastric acidity, and enable controlled and site‐specific release in the intestinal tract.^[^ ^239^ ^]^ Polymers such as hydroxypropyl methylcellulose acetate succinate (HPMCAS) and hydroxypropyl methylcellulose phthalate (HPMCP) have been extensively studied due to their enteric and plasticizing properties, which facilitate the encapsulation of AMPs and pH‐sensitive bioactives.^[^ ^240^, ^241^, ^242^ ^]^ HPMCAS is known to dissolve at pH levels above 5.5, making it suitable for protecting compounds in the stomach and ensuring their release in the upper small intestine. In contrast, HPMCP dissolves at higher pH values (typically above 6.0), allowing for a more distal release. This approach has proven effective in preventing the premature degradation of AMPs and in enhancing their bioavailability, structural stability, antimicrobial activity, and functional performance during gastrointestinal transit.^[^ ^21^ ^]^

### 6.5 Metallic Nanoparticles (MNPs)

Nanotechnology has expanded to a wide range of clinical applications due to the presence of metallic nanoparticles (MNPs), which exhibit unique antimicrobial properties essential for manufacturing new drugs and medical devices.^[^ ^243^ ^]^ Synthesis methods are well described in the literature and are divided into two sets of techniques: those based on the top‐down method and those based on the bottom‐up method. Top‐down techniques include sputtering, laser ablation, lithography, mechanical milling, and thermal decomposition. In contrast, bottom‐up techniques consist of the Turkevich and Brust methods, as well as green synthesis.^[^ ^244^ ^]^

The combination of MNPs with various antimicrobial drugs, such as AMPs, antibiotics, antifungals, and antivirals, can overcome the limitations of using them individually and improve efficacy.^[^ ^245^ ^]^ Conjugated MNPs provide effective delivery of therapeutic drugs, substantially increasing the amount of medication at the action site in specific cells with minimal adverse effects, broadening the spectrum and enhancing drug stabilization. This allows for cellular‐level diagnosis and treatment.^[^ ^199^ ^]^ Conjugated MNPs deliver therapeutic drugs effectively, substantially increasing the concentration of medication at the target site in specific cells with minimal adverse effects, broadening the spectrum of activity, and enhancing drug stabilization.^[^ ^199^ ^]^ However, metallic nanoparticles have disadvantages: they may be toxic due to the reagents used in synthesis, can precipitate and aggregate, and lack biodegradability.^[^ ^246^ ^]^

Nonetheless, AMP‐conjugated MNPs can improve their antimicrobial efficacy while allowing for stabilization, preventing aggregation, and achieving synergistic antibacterial effects.^[^ ^186^ ^]^ Xu et al.^[^ ^247^ ^]^ presented a study on the advantages of nanocarriers based on silver nanoparticles modified with antimicrobial peptides (AMP@PDA@AgNPs) for combating biofilms and resistant bacteria. The results indicated that AMP@PDA@AgNPs had an average size of 200 nm, as determined by DLS and TEM analyses. These nanoparticles showed greater efficacy against gram‐negative bacteria such as *E. coli* and *P. aeruginosa*, as well as gram‐positive bacteria such as *S. aureus*. This study revealed that AMP@PDA@AgNPs inhibited bacterial growth and destroyed biofilms by reducing the expression of genes related to biofilm formation (fimH, lasI, and rhII), resulting in a 50% reduction in gene expression at a concentration of 100 µg mL^−1^. Furthermore, AMP@PDA@AgNPs successfully reduced the thickness and biomass of biofilms. This ability was attributed to the synergistic interaction between antimicrobial peptides and silver nanoparticles, which penetrated bacterial membranes and disrupted their integrity.

Zhen et al.^[^ ^248^ ^]^ developed an innovative nanosystem that combines poly(ε‐caprolactone) (PCL), a biodegradable and hydrophobic polymer that promotes nanoparticle formation, with AMPs, which act as a stabilizing support for AgNPs and provide intrinsic antimicrobial activity. This system, which was based on silver nanoparticles decorated with star‐shaped PCL‐b‐AMP copolymers (AgNPs@PCL‐b‐AMPs), was characterized by an average hydrodynamic diameter of 264.18 nm and a zeta potential of +21.9 mV. The system demonstrated high efficacy against gram‐positive and gram‐negative bacteria, achieving low MIC values ranging from 2 to 8 µg mL^−1^, even against resistant strains such as methicillin‐resistant *S. aureus* (MRSA) and vancomycin‐resistant *Enterococcus* (VRE). The antimicrobial activity of the system is attributed to a multimodal mechanism that includes bacterial membrane disruption, intracellular ion leakage, and the generation of reactive oxygen species (ROS), ultimately leading to bacterial apoptosis. Additionally, in vivo assays confirmed that this nanocarrier significantly reduces bacterial loads in infected organs and does not induce bacterial resistance, even after 21 bacterial passages. This nanocarrier is promising for reducing dosage because of its prolonged effect and ability to address challenges associated with antibiotic resistance.

**Figure 7:**
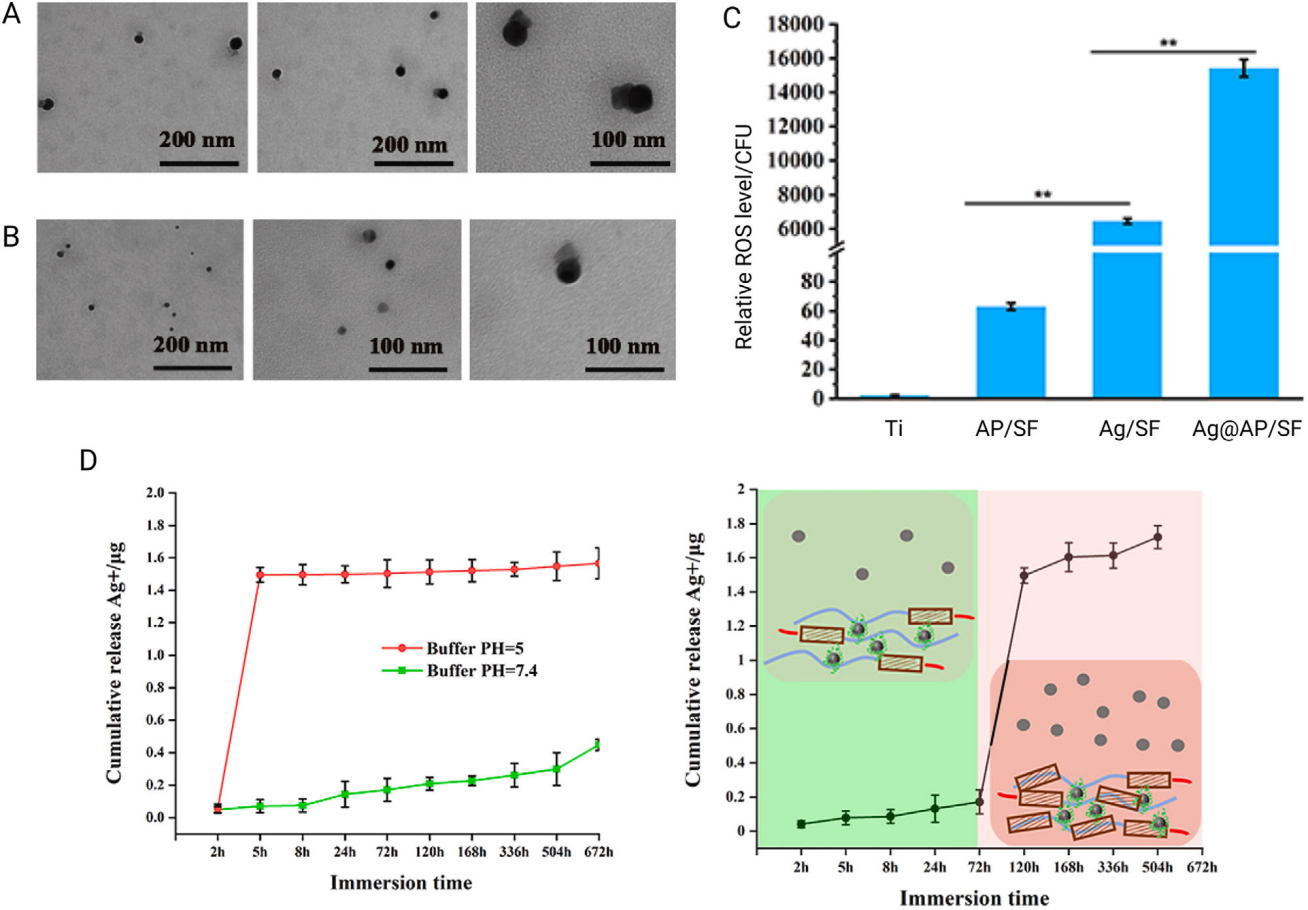
A) AgNPs reduced by SF with an approximate size of 50 nm. B) AgNPs reduced by SF/AMP with a similar size. C) The ROS content in bacteria treated with Ag@AP/SF was greater than that in the other groups, indicating that the formation of AgNP@AMPs promotes ROS production, contributing to enhanced antibacterial activity. D) Cumulative release of Ag⁺ ions from the Ag@AP/SF system under different pH conditions. At pH 5 (red line), Ag⁺ is rapidly released, reaching ≈1.5 µg within the first few hours, whereas at pH 7.4 (green line), the release is slower and sustained, reaching only 0.15 µg over several days. E) Schematic representation of the system's behavior: under neutral conditions (green zone), Ag@AP/SF maintains controlled silver release, whereas in an acidic environment (red zone, simulating bacterial infection), the structure destabilizes, triggering accelerated Ag⁺ release due to silk fibroin transformation, thereby enhancing its pH‐activated antibacterial capability. Reproduced with permission.^[^ ^249^ ^]^ Copyright 2023, Elsevier B.V.

Zhou et al.^[^ ^249^ ^]^ developed a nanocarrier based on AgNPs combined with AMPs and silk fibroin (SF). This system has outstanding results in terms of both antimicrobial activity and osteogenesis. The size of the silver nanoparticles was reduced from 50 nm to approximately 20 nm with the addition of AMPs, improving their dispersion and biological activity (**Figure** **7**A,B). This coating achieved a bacterial inhibition rate of over 99% against *S. aureus* after 21 days because of mechanisms such as membrane destruction, ROS generation, and silver ion release under acidic conditions simulating infection (Figure 7C). Controlled silver ion release increased approximately tenfold in acidic environments (pH 5) compared with neutral conditions (pH 7.4), with sustained release occurring over 28 days (Figure 7D,E). The combination of AMPs with AgNPs reduced toxicity by allowing the use of lower nanoparticle concentrations while maintaining high antimicrobial efficacy. In terms of osteogenic activity, the system promoted the adhesion, proliferation, and osteogenic differentiation of bone marrow stem cells, increasing the expression of alkaline phosphatase, collagen production, and calcium deposition within 28 days. These results highlight that this nanocarrier not only exhibits excellent antimicrobial and osteogenic performance but also effectively adapts to acidic conditions, such as those present in gastric or infectious environments.

### 6.6 Mesoporous Silica Nanoparticles

Mesoporous silica nanoparticles (MSNs) offer adjustable and well‐defined characteristics, including particle and pore size, pore volume, surface area, structural array of pores, and surface functionality.^[^ ^250^, ^251^ ^]^ Most MSNs can be produced through efficient and economical processes by controlling key parameters such as the reagent concentration, temperature, reaction mixture pH, surfactants, and polymers, as well as the types of silica sources used.^[^ ^252^ ^]^ MSNs are synthesized via the self‐assembly of surfactant molecules as templates, which are then condensed around silica precursors and removed to yield a material with cavity networks. These materials feature a uniform pore size distribution from 2 to 50 nm, high pore volume and surface area, and a high density of silanol groups on the surface, making them ideal nanocarriers for designing smart drug delivery nanocarriers.^[^ ^253^ ^]^ AMPs can be conjugated to MSNs or incorporated into their mesopores for controlled release. The antimicrobial efficacy of AMP‐functionalized MSNs is influenced by the AMP loading amount and the nanoparticle porosity, which affects AMP release and impacts bacterial membranes.^[^ ^254^ ^]^

Yu et al.^[^ ^255^ ^]^ highlighted the use of MSN‐based nanocarriers for the delivery of the AMP melittin and antibiotics. These systems offer a multistimuli design that allows for the controlled and efficient release of therapeutic agents under specific stimuli, such as the presence of pathogenic bacteria, heat, or alternating magnetic fields (AMF). The nanoparticles used have distinct sizes, with the host particles displaying hydrated diameters between 230–350 nm, whereas the guest particles have sizes ranging from 70–85 nm after functionalization. Additionally, they exhibit different zeta potentials, with values of +48.4 mV for the host particles and +31.5 mV for the guest particles after supramolecular assembly. The system demonstrated synergistic eradication of pathogenic biofilms (*P. aeruginosa*) by coreleasing the AMP melittin and the antibiotic ofloxacin. In vitro experiments revealed that the assemblies achieved complete biofilm elimination (97%) and 100% bacterial cell death under AMF, outperforming free drugs or individual nanoparticles. Furthermore, its biocompatibility was confirmed, as it did not cause toxicity in mammalian cells such as NIH3T3 and 293T cells. These results demonstrate that this platform is effective in overcoming multidrug resistance, penetrating resistant biofilms, and delivering specific therapeutic combinations with high precision and safety.

Additionally, Zhao et al.^[^ ^256^ ^]^ synthesized MSNs as a delivery system for the peptide T7E21R‐HD5, a designed variant of the human defensin HD5. These nanoparticles, with an average size of 60 nm and an initial Z potential of −14.6 mV, proved to be an ideal platform for the electrostatic attraction of positively charged peptides. The coating of MSNs with succinylated casein (SCN) protected the peptide from enzymatic degradation in the acidic environment of the stomach and ensured controlled release in the intestine, triggered by the presence of trypsin. This design overcomes the traditional limitations of AMPs, such as instability and rapid degradation under physiological conditions. The MSN@T7E21R‐HD5 system enhanced antimicrobial activity against MDR *E. coli*. Compared with the free peptide, MSNs increased the permeability of the bacterial outer and inner membranes, resulting in an MIC that was 32 times lower. SEM analyses revealed severe damage to the bacterial membrane, including fragmentation and cytoplasmic content loss. In vivo studies demonstrated that the MSN@T7E21R‐HD5@SCN system was effective at eliminating MDR *E. coli* and reducing intestinal inflammation by lowering the levels of inflammatory cytokines such as TNF‐α, IL‐1β, and MMP‐9. This study highlights the capacity of MSNs to protect AMPs in the hostile environment of the stomach.

Ma et al.^[^ ^257^ ^]^ highlighted the advantages of using nanocarriers such as MSNs modified with ovotransferrin‐derived AMP (OVTp12) to load and target antibiotics toward specific bacteria. These nanoparticles are approximately 79–98 nm in size, and the zeta potential shifts from −18.9 mV to 12.9 mV after modification with OVTp12. Compared with unmodified nanoparticles, MSNs modified with OVTp12 and loaded with gentamicin (MSNs@OVTp12@Gen) inhibited *E. coli* growth both in vitro and in vivo. This resulted in larger inhibition zones in the culture media and a significant reduction in bacterial levels in the infected tissues. Additionally, controlled release of the antibiotic in acidic environments was demonstrated, which is advantageous at bacterial infection sites. In murine models, treatment with MSNs@OVTp12@Gen led to a reduction in inflammatory cytokines (IL‐1β, IL‐6, and TNF‐α) and improved survival rates against bacterial infections, with no toxic effects on major organs.

## 7 Conclusion and Perspectives

Conventional treatments for gastrointestinal infections face multiple challenges, including difficulties in achieving optimal drug concentrations at the infection site, the increasing proliferation of multidrug‐resistant bacteria, and the instability of certain compounds coupled with toxicity risks. Furthermore, the overuse of antibiotics can disrupt the intestinal microbiota and trigger dysbiosis, which exacerbates clinical complications and diminishes the effectiveness of existing therapies. In the search for effective solutions, nanotechnology has emerged as a promising avenue by integrating with AMPs. Beyond enabling controlled release and protecting AMPs from enzymatic degradation, these platforms bring additional advantages, such as enhanced bioavailability, the ability to target specific areas within the GIT, and the incorporation of mucoadhesive functionalities. This approach not only amplifies the effectiveness of AMPs but also helps preserve beneficial microbiota by minimizing exposure in non‐target regions and curbing the emergence of resistance. Despite these advances, implementing such technologies in clinical practice still demands overcoming several hurdles. Chief among them is deepening our understanding of how AMPs and nanoparticles interact with the intestinal ecosystem, as well as refining large‐scale production processes to ensure both economic viability and accessibility, especially in resource‐limited settings. Additionally, it is essential to expand the number of in vivo studies and clinical trials that verify the efficacy and safety of these technological solutions. Looking ahead, the convergence of nanotechnology, molecular biology, and precision medicine will enable the design of nanocarriers with selective properties capable of targeting specific pathogens without substantially affecting the rest of the intestinal flora. Such a collaborative vision requires a concerted effort among scientists from diverse fields, healthcare professionals, and regulatory authorities to establish clear standards for quality, biosafety, and effectiveness, ensuring the responsible use of these therapies. Ultimately, incorporating antimicrobial peptides into nanotechnological platforms represents a potential paradigm shift in the management of gastrointestinal infections. The prospect of more targeted, efficient, and less toxic treatments paves the way for a new generation of therapeutic solutions, the ones that not only eradicate pathogens more effectively but also foster long‐term intestinal health.

## Conflict of Interest

The authors declare no conflict of interest.

## Author Contributions

**Christian S. Carnero Canales**: Writing – review & editing, Writing – original draft, Validation, Investigation, Conceptualization. **Jessica Ingrid Marquez Cazorla**: Writing – original draft, Visualization, Validation, Investigation, Formal analysis. **Renzo Marianito Marquez Cazorla**: Writing – original draft, Visualization, Validation, Methodology, Investigation, Formal analysis. **Vanderson de Jesus Silva, Laura Maria Duran Gleriani Primo, Maura Jennifer Martinez Morales and Uner Josseph Pinto Apaza**: Writing – original draft, Investigation, Formal analysis **Cesar Augusto Roque‐Borda**: Writing – review & editing, Visualization, Validation, Supervision. **Rafael Miguel Sábio**: Writing – review & editing, Writing – original draft, Visualization, Validation. **Hélder A. Santos**: Writing – review & editing, Validation, Supervision, Resources, Project administration, Funding acquisition, Conceptualization. **Fernando Rogério Pavan**: Writing – review & editing, Visualization, Validation, Supervision, Resources, Project administration, Funding acquisition, Conceptualization.
